# Heterochromatin suppresses gross chromosomal rearrangements at centromeres by repressing Tfs1/TFIIS-dependent transcription

**DOI:** 10.1038/s42003-018-0251-z

**Published:** 2019-01-11

**Authors:** Akiko K. Okita, Faria Zafar, Jie Su, Dayalini Weerasekara, Takuya Kajitani, Tatsuro S. Takahashi, Hiroshi Kimura, Yota Murakami, Hisao Masukata, Takuro Nakagawa

**Affiliations:** 1 Department of Biological Sciences, Graduate School of Science, Osaka University, 1-1 Machikaneyama, Toyonaka, Osaka, 560-0043 Japan; 2 Department of Chemistry, Faculty of Science, Hokkaido University, Sapporo, Hokkaido 060-0810 Japan; 3 Cell Biology Center, Institute of Innovative Research, Tokyo Institute of Technology, 4259 Nagatsuta, Midori-ku, Yokohama, Kanagawa 226-8503 Japan; 4 Present Address: Department of Molecular Biology and Genetics, Cornell University, 526 Campus Road, Ithaca, NY 14853 USA; 5 Present Address: Department of Biology, Faculty of Science, Kyushu University, 744 Motooka, Nishi-ku, Fukuoka, 819-0395 Japan

## Abstract

Heterochromatin, characterized by histone H3 lysine 9 (H3K9) methylation, assembles on repetitive regions including centromeres. Although centromeric heterochromatin is important for correct segregation of chromosomes, its exact role in maintaining centromere integrity remains elusive. Here, we found in fission yeast that heterochromatin suppresses gross chromosomal rearrangements (GCRs) at centromeres. Mutations in Clr4/Suv39 methyltransferase increased the formation of isochromosomes, whose breakpoints were located in centromere repeats. H3K9A and H3K9R mutations also increased GCRs, suggesting that Clr4 suppresses centromeric GCRs via H3K9 methylation. HP1 homologs Swi6 and Chp2 and the RNAi component Chp1 were the chromodomain proteins essential for full suppression of GCRs. Remarkably, mutations in RNA polymerase II (RNAPII) or Tfs1/TFIIS, the transcription factor that facilitates restart of RNAPII after backtracking, specifically bypassed the requirement of Clr4 for suppressing GCRs. These results demonstrate that heterochromatin suppresses GCRs by repressing Tfs1-dependent transcription of centromere repeats.

## Abstract (web summary)

Akiko Okita et al. demonstrate that heterochromatin suppresses gross chromosomal rearrangements by repressing Tfs1/TFIIS-dependent transcription of repetitive sequences. This study underscores the role of heterochromatin for maintaining chromosome stability.

## Introduction

Repetitive DNA elements such as centromere repeats and transposable elements are prevalent in eukaryotic genomes and occupy at least 50% of the human genome^1^. The presence of repetitive elements is a threat to genome stability. Recombination events such as crossover and break-induced replication (BIR) between repetitive elements give rise to gross chromosomal rearrangements (GCRs), which cause cell death and genetic diseases including cancer^2,3^. Most of the repetitive elements, including centromere repeats, are present in heterochromatin domains and transcriptionally silenced^4^. Transcriptional de-repression of repetitive elements (also called satellite DNA) has been observed in a variety of cancers^5,6^, suggesting a link between GCRs and transcription of repetitive elements.

Heterochromatin is marked by histone H3 lysine 9 (H3K9) methylation that is catalyzed by specific methyltransferases such as fission yeast Clr4 and mammalian Suv39^7^. A *clr4* deletion increases RNA polymerase II (RNAPII) localization and de-represses transcription at centromere repeats^8^, demonstrating that H3K9 methylation causes transcriptional silencing. The H3K9 methylation mark is recognized by chromodomain proteins such as Heterochromatin Protein 1 (HP1)^9,10^, which creates phase-separated compartments in the nucleus^11^. RNA interference (RNAi) that utilizes small RNAs mediates heterochromatin assembly^12,13^. In fission yeast, the RNA-induced transcriptional silencing (RITS) complex, which consists of small RNAs, Ago1, Chp1, and Tas3, localizes to the centromeres through the Chp1 chromodomain protein and Ago1 that captures small RNAs^8,14–18^. The RITS complex recruits the Clr4-Rik1-Cul4 (CLRC) complex and facilitates H3K9 methylation at the centromeres. In addition to RNAi, the exosome-dependent RNA degradation also contributes to transcriptional silencing. Cid14 is an essential component of the Trf4/Air2/Mtr4 polyadenylation (TRAMP) complex that promotes exosome-dependent degradation of RNAs including centromere transcripts^19^. Mlo3 RNA-binding protein, the homolog of budding yeast Yra1 and mammalian Aly/REF, is required for the export of poly(A)^+^ RNA from the nucleus^20–22^. Yra1 directly binds to the C-terminal domain of RNAPII^23^, facilitating the transcription-coupled loading of RNA export factors. Like RNAPII, Mlo3 localizes to the gene body of the euchromatin, and it binds to centromere repeats in the absence of Clr4^24^. Mlo3 also interacts with Cid14 and facilitates the exosome-dependent RNA degradation^24^. Loss of either Mlo3 or Cid14 restores H3K9 methylation in *ago1∆* cells^25^, probably via the recruitment of the CLRC complex to non-degraded nascent transcripts at the centromeres.

Centromeres play an essential role in the correct segregation of chromosomes. Centromeres comprise species-specific centromere repeats in many eukaryotes and are one of the fragile sites of the chromosomes. Chromosome breakages frequently occur at centromeres during tumorigenesis, and the centromere sequence and position change rapidly during the process of evolution^26,27^. Robertsonian translocation that occurs around centromeres of acrocentric chromosomes is the most common type of chromosomal abnormality observed in humans (1 per 1000 individuals)^28^. The formation of isochromosomes, whose arms are mirror images of each other, is mediated by inverted repeats at the centromeres in *Schizosaccharomyces pombe* and *Candida albicans*^29,30^. Heterochromatin ensures sister chromatid cohesion at the centromeres^31^ and prevents incorrect attachment of spindle microtubules to kinetochores^32^. However, heterochromatin is not always formed at the centromeres: heterochromatin assembly is sometimes lacking at the centromeres that are devoid of repetitive elements^33–35^. Heterochromatin may have an important role especially when the centromeres consist of repetitive elements.

Heterochromatin plays an important role in the maintenance of genome integrity. Suv39 knockout mice exhibit chromosome aneuploidy and predisposition to develop cancer^36^. Loss of H3K9 methylation in *Caenorhabditis elegans* increases instability of repetitive elements probably through the formation of RNA:DNA hybrids^37^. In fission yeast, heterochromatin appears to prevent replication fork collapse and DNA recombination at the centromeres^38,39^. Heterochromatin prevents DNA double-strand break formation at the centromeres in meiosis^40^. However, how heterochromatin affects GCRs between centromere repeats remains elusive.

Here, we found that heterochromatin suppresses GCRs at the centromeres of fission yeast. Deletion of Clr4 increased the formation of isochromosomes, whose breakpoints were located in centromere repeats. Amino acid substitutions in H3K9 (i.e., H3K9A and H3K9R) also increased GCR rates, suggesting that Clr4 suppresses centromeric GCRs through H3K9 methylation. Mutations in the HP1 homologs, Swi6 and Chp2, and the RNAi component Chp1 synergistically increased the GCR rate, showing that both HP1 and RNAi machinery are required to suppress GCRs. Mutations in the C-terminal domain (CTD) of RNAPII impaired chromatin binding of RNAPII and reduced GCRs in *clr4∆* cells. Tfs1/TFIIS is the transcription factor that facilitates restart of transcription elongation when RNAPII is paused and backtracked on template DNA^41,42^. Strikingly, *tfs1∆* specifically bypassed the requirement of Clr4 for GCR suppression, without changing chromatin binding levels of RNAPII. These data demonstrate that heterochromatin suppresses GCRs by repressing Tfs1/TFIIS-dependent transcription of repetitive sequences.

## Results

### Clr4 suppresses GCRs through H3K9 methylation

**Fig. 1:**
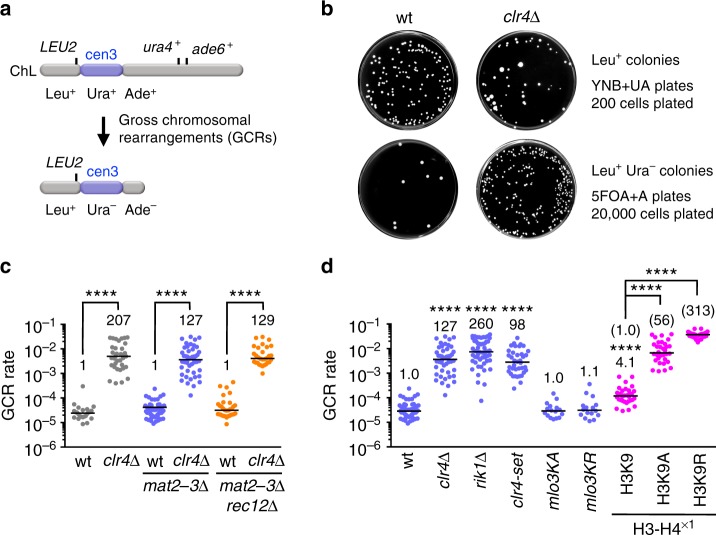
Clr4 methyltransferase suppresses gross chromosomal rearrangements (GCRs) through H3K9 methylation. **a** Illustration of an extra-chromosome ChL. Positions of *LEU2*, *ura4*^+^, *ade6*^+^, and centromere 3 (cen3) are indicated. When GCRs associated with the loss of *ura4*^+^ and *ade6*^+^ take place, Leu^+^ Ura^+^ Ade^+^ cells become Leu^+^ Ura^–^ Ade^–^ cells. **b** Wild-type and *clr4∆* strains (TNF5676 and 5702, respectively) grown in EMM + UA were plated onto YNB + UA (2 × 10^2^ cells) and 5FOA + A (2 × 10^4^ cells) media to count Leu^+^ and Leu^+^ Ura^–^ colonies, respectively. Plates were incubated at 30 °C for 6–9 days. wt, wild type. **c** GCR rates of wild-type, *clr4∆*, *mat2-3∆*, *mat2-3∆ clr4∆*, *mat2-3∆ rec12∆*, and *mat2-3∆ rec12∆ clr4∆* strains (TNF3896, 5440, 5676, 5702, 5701, and 5766, respectively). Each dot represents the GCR rate determined using a single colony formed on EMM + UA plates in scatter plots. Lines represent the median. The GCR rate relative to that of the wild-type *clr4*^+^ strain is indicated on the top of each column. Statistical significance of differences between pairs of strains was determined using the two-tailed Mann–Whitney test. ****P < 0.0001. **d** GCR rates of wild-type, *clr4∆*, *rik1∆*, *clr4-set*, *mlo3KA*, *mlo3KR*, H3K9, H3K9A, and H3K9R strains in the *mat2-3∆* background (TNF5676, 5702, 6121, 6958, 6155, 6157, 5738, 6223, and 5802, respectively). The GCR rate relative to that of wild type is indicated on the top of each column. In the cases of H3K9, H3K9A, and H3K9R strains, the GCR rate relative to that of the wild-type H3K9 strain is also shown in parentheses. Statistical significance of differences relative to wild type (the top of each column), and of differences between pairs of strains was determined using the two-tailed Mann–Whitney test

Clr4 is essential for H3K9 methylation in fission yeast. To understand the role of heterochromatin in genome stability, we disrupted the *clr4* gene and determined the rate of spontaneous GCRs^29^. We detected otherwise lethal GCRs in haploid cells, using an extra-chromosome ChL derived from chromosome 3 (chr3)^29,43^ (Fig. 1a). Cells harboring ChL (Leu^+^ Ura^+^ Ade^+^) were grown in the minimum medium supplemented with uracil and adenine (EMM + UA), and then plated onto YNB + UA and YNB supplemented with 5-fluoroorotic acid and adenine (5FOA + A) to count Leu^+^ and Leu^+^ Ura^–^ colonies, respectively. The *clr4∆* strain produced slightly fewer Leu^+^ colonies than wild type (Fig. 1b), probably due to high incidence of chromosome loss. However, *clr4∆* formed more Leu^+^ Ura^–^ colonies than wild type (Fig. 1b). Leu^+^ Ura^–^ colonies were replicated onto EMM + U plates to test whether they are Ade^+^ or Ade^–^ (see Methods). Almost all Leu^+^ Ura^–^ colonies were Leu^+^ Ura^–^ Ade^–^. Using the numbers of Leu^+^ and Leu^+^ Ura^–^ Ade^–^ cells (see Methods), we determined the GCR rate by means of a fluctuation analysis^44^ and found that it was strongly increased by *clr4∆* (Fig. 1c, gray dots). Because *clr4∆* de-represses the silent mating-type locus *mat2P-mat3M* and occasionally forms diploid cells^45^, *clr4∆* might increase GCRs by potentiating expression of the meiotic genes including Rec12/Spo11, which creates DNA double-strand breaks^40,46^. However, *clr4∆* increased GCR rate even in the absence of *mat2P-mat3M* (Fig. 1c, blue dots) and *rec12* (Fig. 1c, orange dots). These results show that Clr4 suppresses spontaneous GCRs in mitotic cells. Nevertheless, *mat2-3∆* strains were used hereafter to exclude any possible effects of the silent mating-type locus de-repression.

Next, we sought to elucidate how Clr4 suppresses GCRs. Rik1, a component of the CLRC complex is required for the localization of Clr4 to heterochromatin regions^8,47^. Like *clr4∆*, *rik1∆* increased GCR rate (Fig. 1d, blue dots), suggesting that chromatin localization of Clr4 is required to suppress GCRs. The R/HφφNH (*φ* = hydrophobic residues) motif in the SET domain constitutes the binding site of S-adenosyl-L-methionine (SAM), which is essential for the methyl transfer^7,48,49^. It has been shown that single amino acid substitutions in the SET domain impair methyltransferase activity of recombinant Clr4 in vitro, but the mutant strains show residual levels of H3K9 methylation at centromeres in vivo^9^. To examine if the methyltransferase activity of Clr4 is required to suppress GCRs, we introduced alanine substitutions at the three evolutionally conserved residues, R406, N409, and H410, in the R/HφφNH motif of the SET domain (Supplementary Fig. 1a). We prepared extracts from the yeast that expressed wild-type Flag-Clr4 or mutant Flag-Clr4-set protein from the native promoter, performed Western blotting using anti-Flag antibodies, and found that the *clr4-set* mutation only slightly reduces the protein level (Supplementary Fig. 1b). Chromatin immunoprecipitation (ChIP) showed that *clr4-set* completely abolished di-methylation and tri-methylation of H3K9 (H3K9me2 and H3K9me3, respectively) at the centromeres (Supplementary Fig. 1c). Like *clr4∆*, *clr4-set* increased GCR rate (Fig. 1d, blue dots). Clr4 methyltransferase has other targets including Mlo3^24,50^, in addition to histone H3. Neither alanine (*mlo3KA*) nor arginine (*mlo3KR*) substitution in Mlo3 methylation sites significantly changed GCR rate (*P* = 0.93 and 0.73, respectively) (Fig. 1d, blue dots). We examined the effect of H3K9 mutations in the H3-H4^×1^ strain background where two out of three H3-H4 genes in the genome have been eliminated^51^. Reducing the copy number of H3-H4 genes by itself slightly increased GCR rate (Fig. 1d, magenta dots). Either alanine (H3K9A) or arginine (H3K9R) substitution further increased GCR rate, showing the importance of H3K9 in GCR suppression. These results suggest that Clr4 suppresses GCRs through H3K9 methylation.

### Clr4 and Rik1 suppress isochromosome formation at centromeres

**Fig. 2:**
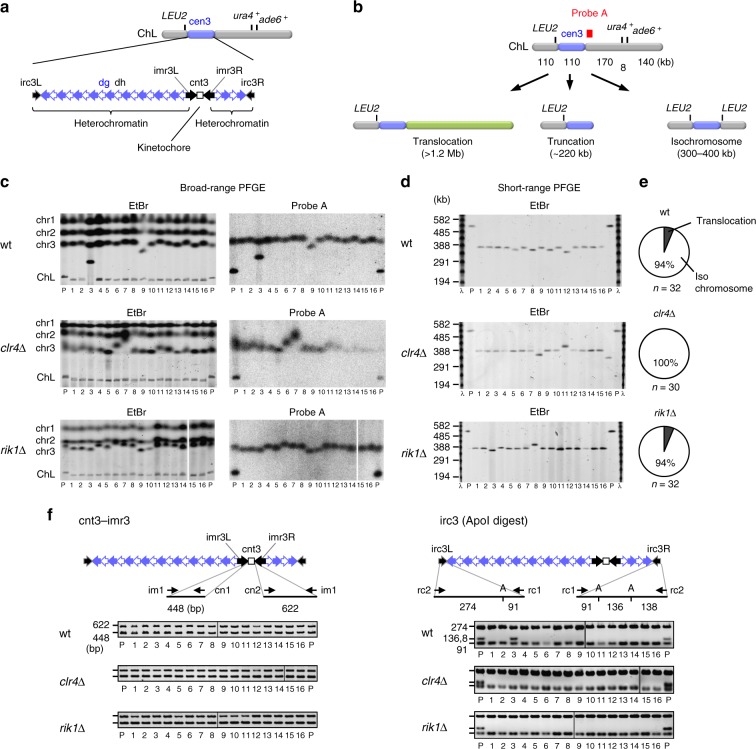
Clr4 and Rik1 suppress the formation of isochromosomes whose breakpoints are located in centromere repeats. **a** Repetitive sequences in cen3 of ChL are shown. Units of centromere repeats are indicated as arrows. **b** Illustration of the gross chromosomal rearrangement (GCR) products that have lost *ura4*^+^ and *ade6*^+^ from ChL: translocation, truncation, and isochromosome. The position of probe A used in Southern hybridization is indicated as filled box. **c** Chromosomal DNAs of wild-type, *clr4∆*, and *rik1∆* strains (TNF5676, 5702, and 6121, respectively) were separated by broad-range pulse field gel electrophoresis (PFGE) and stained with ethidium bromide (EtBr). Positions of chr1, chr2, chr3, and ChL (5.7, 4.6, ~3.5, and 0.5 Mb, respectively) in the parental strain are indicated on the left of the panel. DNAs were transferred onto a nylon membrane and hybridized with probe A. P, Parental. **d** Chromosomal DNAs were separated by short-range PFGE and stained with EtBr. Sizes of the λ DNA ladder are indicated on the left of the panel. **e** Pie charts depict proportions of different types of GCRs. **f** Breakpoints were determined by PCR reactions using GCR products recovered from agarose gel. Both sides of cnt3–imr3 junctions were amplified in the reaction containing im1, cn1, and cn2 primers. irc3L and irc3R were amplified using rc1 and rc2 primers, and the PCR products were digested by ApoI and separated by agarose gel electrophoresis. A, ApoI. Uncropped images of depicted gels and blots are shown in Supplementary Figs. 10 and 11

Kinetochore chromatin, characterized by the centromere-specific H3 variant CENP-A, is formed on the central sequence (cnt), whereas heterochromatin assembles on the flanking inverted repeats (imr, dg, dh, and irc) (Fig. 2a)^52^. Loss of *ura4*^+^ and *ade6*^+^ from ChL results either from translocation, truncation, or isochromosome formation (Fig. 2b)^29,53,54^. Isochromosomes are produced by recombination between inverted repeats at the centromeres. To determine whether heterochromatin affects GCRs at the centromeres, chromosomal DNAs of parental and independent GCR clones of wild-type, *clr4∆*, and *rik1∆* strains were prepared in agarose plugs, separated by broad-range pulse field gel electrophoresis (PFGE), and stained with ethidium bromide (EtBr) (Fig. 2c and Supplementary Fig. 2a). In wild type, among the 32 GCR products examined, there were two translocations larger than the parental ChL (Fig. 2c, wt #3; Supplementary Fig. 2a, wt #27). Other GCR products were smaller than the parental ChL. The parental ChL was detected, but the small GCR products were not detected by Southern blotting using probe A that hybridizes to the right side of cen3 (Fig. 2b, c and Supplementary Fig. 2a), suggesting that they have completely lost the right arm of the parental ChL. The size of the truncated chromosome that have lost the entire region of the right arm would be ~220 kb (Fig. 2b). Short-range PFGE showed that small GCR products were in the range of 300–400 kb but not ~220 kb (Fig. 2b, d, and Supplementary Fig. 2b), indicating that they were isochromosomes but not truncations. Variable sizes of individual isochromosomes may be explained by the difference in the copy number of dg and dh repeats^29^. The total length of cen3 becomes longer in isochromosomes when recombination between a pair of inverted repeats (imr3, dg, or dh) occurs and the right side of cen3 gains an increased number of dg and dh repeats^29^. Around 6% of GCR products were translocations in this study, where the minimal medium was utilized (Fig. 2e). In contrast, in our previous study, where we used rich medium, ~50% of GCR products were translocations^53^. The difference may be due to severe growth disadvantage of the cells containing a translocation in the minimal medium. Similar to the case with wild type (30 out of 32), most of the GCR products formed in *clr4∆* (30 out of 30) and *rik1∆* (30 out of 32) strains were isochromosomes (Fig. 2e, *P* > 0.4, the two-tailed Fisher’s exact test). Given the high rates of GCRs in *clr4∆* and *rik1∆* strains (Fig. 1d), these data show that Clr4 and Rik1 suppress GCRs especially the isochromosome formation.

To see whether breakpoints are located in centromere repeats, GCR products were recovered from agarose gel and analyzed by PCR. In all samples examined, both sides of cnt3–imr3 junctions were amplified (Fig. 2f and Supplementary Fig. 2c, cnt3–imr3). However, ApoI restriction fragments (136 and 138 bp) of the irc3 PCR product that are indicative of the right side of irc3 (irc3R) were not detected in all isochromosomes (Fig. 2f and Supplementary Fig. 2c, irc3 (ApoI digest)). We further confirmed that the boundary between cen3 and arm regions was specifically missing on the right side in all isochromosomes (Supplementary Fig. 2d). Together with the absence of the probe A region in isochromosomes, these results show that Clr4 and Rik1 suppress the formation of isochromosomes whose breakpoints are located in centromere repeats.

### Chromodomain proteins are required for GCR suppression

**Fig. 3:**
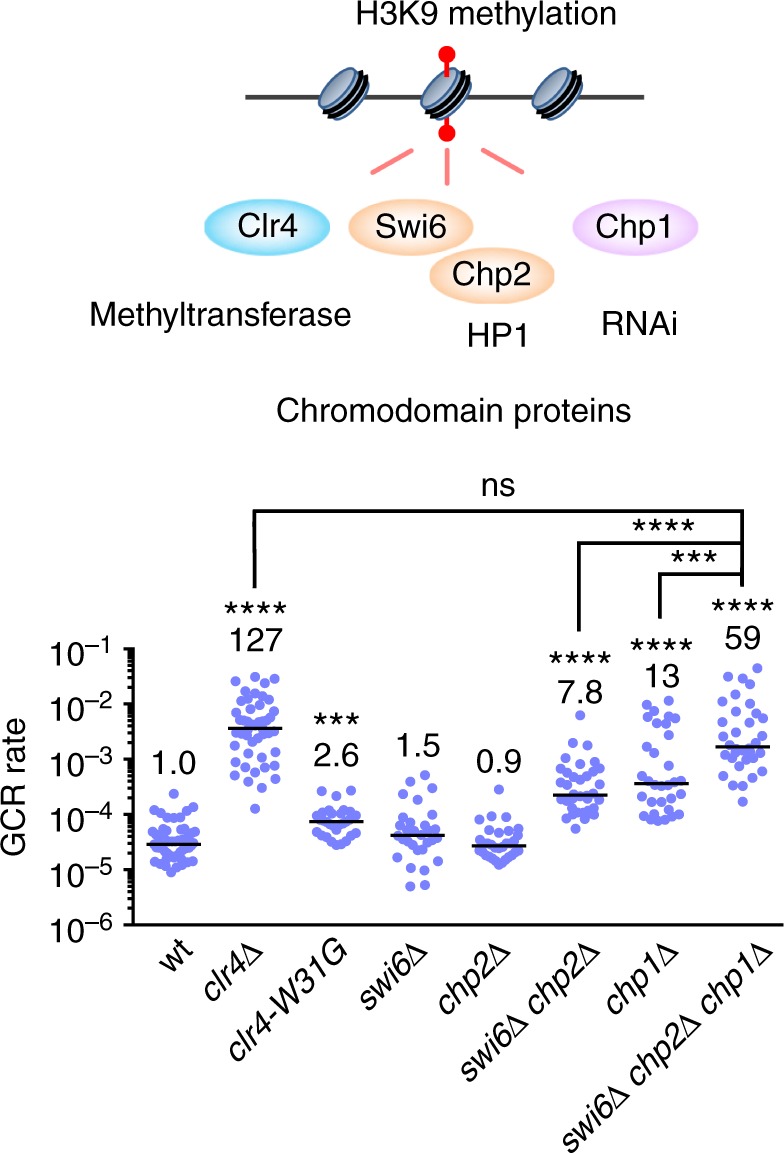
Both HP1 homologs, Swi6 and Chp2, and the RNAi component Chp1 are the chromodomain proteins essential for full suppression of gross chromosomal rearrangements (GCRs). The chromodomain proteins Clr4, Swi6, Chp2, and Chp1 that bind to H3K9 methylation marks are illustrated. GCR rates of wild-type, *clr4∆*, *clr4-W31G, swi6∆*, *chp2∆, swi6∆ chp2∆, chp1∆*, and *swi6∆ chp2∆ chp1∆* strains (TNF5676, 5702, 6012, 5706, 5685, 5900, 5708, and 6151, respectively) are shown. The two-tailed Mann-Whitney test. ***P < 0.001, ****P < 0.0001; ns, not significant

Clr4, Swi6, Chp2, and Chp1 bind to H3K9me2 and H3K9me3 through the chromodomain^9,10,55–57^ (Fig. 3). H3K9me2 and H3K9me3 are present at similar levels in chromatin-bound histones^58^. RNAi-dependent transcriptional gene silencing occurs on H3K9me2 chromatin. Transition from H3K9me2 to H3K9me3 depends on Clr4 chromodomain and is required for stable binding of Swi6 to nucleosomes^58^. To identify chromodomain proteins important for GCR suppression, we determined GCR rates of the mutant strains of chromodomain proteins (Fig. 3). It has been shown that *clr4-W31G* in Clr4 chromodomain impairs its centromere localization and reduces H3K9me3 but not H3K9me2 levels^9,57,58^ (Supplementary Fig. 1a). *clr4-W31G* only slightly increased GCR rate compared to *clr4∆*, indicating that H3K9me3 plays a minor role in GCR suppression. Neither *swi6∆* nor *chp2∆* significantly affected GCR rate (*P* = 0.08 and 0.76, respectively). A previous study has also shown that *swi6∆* does not significantly increase GCRs^38^. However, the *swi6∆ chp2∆* double mutation increased GCR rate, showing that Swi6 and Chp2 redundantly suppress GCRs. Note that GCR rate of *clr4∆* is 16-fold higher than that of *chp2∆ swi6∆* (*P* < 0.0001), indicating that H3K9 methylation suppresses GCRs only partly through HP1 homologs. Deletion of Chp1, the chromodomain subunit of the RITS complex, increased GCR rate, suggesting that RNAi machinery is required for GCR suppression. *swi6∆ chp2∆* and *chp1∆* synergistically increased GCR rate to the level similar to that of *clr4∆*. Collectively, these results demonstrate that both HP1 homologs and RNAi component Chp1 are the chromodomain proteins that are essential for full suppression of GCRs.

### RNAi machinery is required for GCR suppression at centromeres

**Fig. 4:**
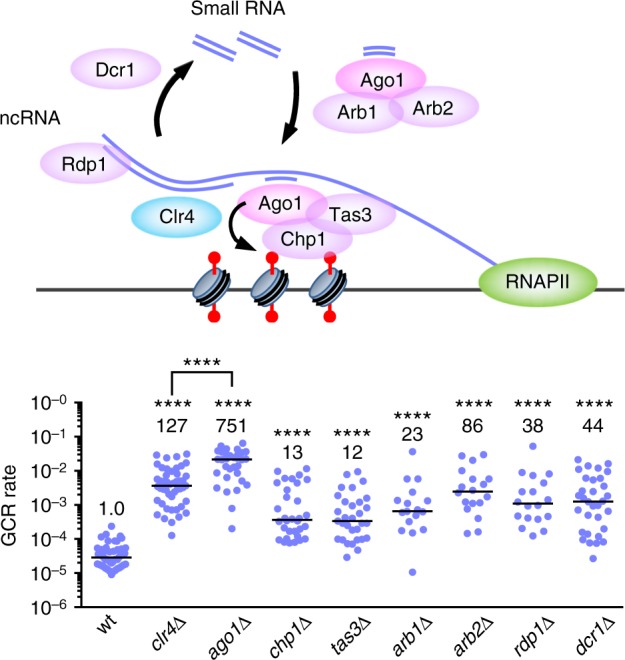
RNAi machinery plays an essential role to suppress gross chromosomal rearrangements (GCRs) at the centromeres. Illustrated is the RNAi system that utilizes small RNAs and facilitates H3K9 methylation at the centromeres. GCR rates of wild-type, *clr4∆*, *ago1∆*, *chp1∆*, *tas3∆*, *arb1∆*, *arb2∆, rdp1∆*, and *dcr1∆* strains (TNF5676, 5702, 5688, 5708, 7335, 7337, 7331, 7333, and 5687, respectively) are shown. The two-tailed Mann–Whitney test. ****P < 0.0001. ncRNA, noncoding RNA. *arb2∆* caused a higher rate of GCRs than *arb1∆* (*P* < 0.05), suggesting that Arb2 has an Arb1-independent function to suppress GCRs

The RNA-directed RNA polymerase Rdp1 creates double-stranded RNAs from noncoding RNAs transcribed from centromere repeats^59^. Dcr1 cleaves double-stranded RNAs to produce small RNAs. Loading of small RNAs onto Ago1 occurs in the Argonaute small interfering RNA chaperon (ARC) complex that contains Ago1, Arb1, and Arb2^60^. Then, Ago1 bound to small RNAs forms the RITS complex with Chp1 and Tas3, and localizes to the centromeres through Chp1 and through base pairing between small RNAs and nascent transcripts at the centromeres^14,16^ (Fig. 4). To establish whether RNAi machinery is required to suppress GCRs at the centromeres, we disrupted these RNAi factors and determined their GCR rates (Fig. 4). Interestingly, *ago1∆* increased GCR rate even greater than *clr4∆*, suggesting that Ago1 not only facilitates H3K9 methylation but also plays some other role to suppress GCRs. GCR rate of *ago1∆* was higher than those of *chp1∆, tas3∆*, *arb1∆*, and *arb2∆* (*P* ≤ 0.0002), suggesting that Ago1 suppresses GCRs partly through the formation of ARC and RITS complexes. GCR rate of *ago1∆* was also higher than those of *rdp1∆* and *dcr1∆* (*P* < 0.0001), probably due to a Dcr1-independent pathway of small RNA production that uses the exosome^61^. Analysis of GCR products formed in *ago1∆* cells showed that most of them (15 out of 16) were isochromosomes whose breakpoints were located in centromere repeats (Supplementary Fig. 3). These results show that RNAi machinery plays an essential role in GCR suppression at the centromeres.

### Ago1 represses RNAPII chromatin binding and GCRs at centromeres

**Fig. 5:**
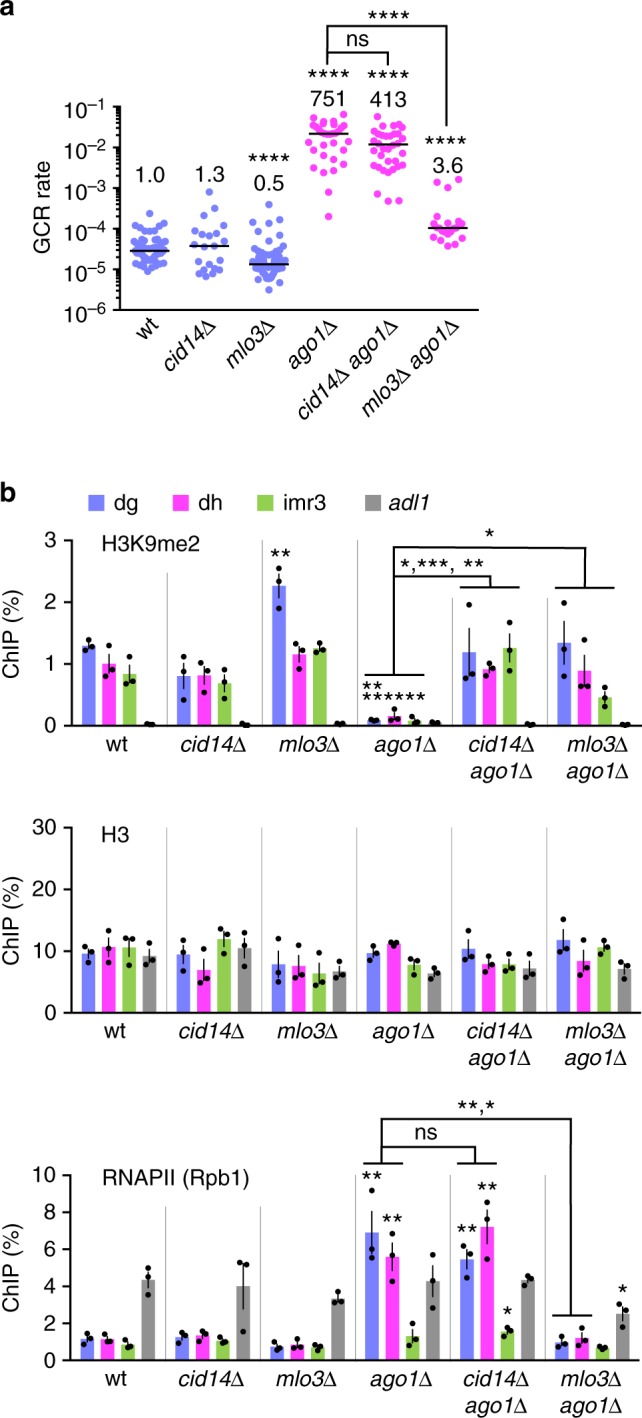
*mlo3∆* but not *cid14∆* reduces RNAPII chromatin binding and suppresses gross chromosomal rearrangements (GCRs) at the centromeres in *ago1∆* cells. **a** GCR rates of wild-type, *cid14∆, mlo3∆*, *ago1∆, cid14∆ ago1∆*, and *mlo3∆ ago1∆* strains (TNF5676, 6153, 5764, 5688, 6411, and 6188, respectively). The two-tailed Mann–Whitney test. ****P < 0.0001; ns, not significant. **b** Chromatin immunoprecipitation (ChIP) analysis was performed to determine H3K9me2, H3 and RNAPII (Rpb1) levels at centromere repeats (dg, dh, and imr3) and at a non-centromeric region of chr2 (*adl1*) in wild-type, *cid14∆, mlo3∆*, *ago1∆, cid14∆ ago1∆*, and *mlo3∆ ago1∆* strains (TNF5921, 6276, 5923, 5922, 6550, and 6210, respectively). DNA levels were quantified by real-time PCR, and percentages of input DNA were obtained. Data are presented as the mean ± s.e.m. from three biologically independent experiments. Dots represent individual measurements from distinct samples. Statistical significance of differences relative to wild type (top of bars), and of differences between pairs of mutant strains was determined using the two-tailed Student’s *t*-test. *P < 0.05, **P < 0.01, ***P < 0.001

To examine whether Ago1 suppresses GCRs only via H3K9 methylation or not, we took advantage of *cid14∆* and *mlo3∆* mutations that restore H3K9me2 levels in *ago1∆* cells^24,25^. *cid14∆* did not significantly change GCR rate in *ago1∆* cells (Fig. 5a, magenta dots, *P* = 0.14). Most of the GCR products formed in *cid14∆ ago1∆* cells were isochromosomes whose breakpoints were located in centromere repeats (14 out of 16 samples) (Supplementary Fig. 4). These results show that the restoration of H3K9me2 levels by *cid14∆* is not sufficient to suppress centromeric GCRs in *ago1∆* cells (also see below). In contrast to *cid14∆*, *mlo3∆* reduced GCR rate in *ago1∆* cells (Fig. 5a, magenta dots).

To find the difference between *cid14∆* and *mlo3∆*, we performed ChIP experiments and determined H3K9me2 and H3 levels at centromere repeats (dg, dh, and imr3) and at a non-centromere region (*adl1*). In wild type, H3K9me2 was specifically detected at dg, dh, and imr3, but not at *adl1* (Fig. 5b, H3K9me2). As expected, *ago1∆* reduced H3K9me2 level at centromere repeats, and both *cid14∆* and *mlo3∆* restored it^24,25^. Similar levels of H3 were observed in all strains examined (Fig. 5b, H3), showing that the mutations affect histone modification rather than nucleosome occupancy. Because Mlo3 is involved in transcription, as well as RNA export and degradation^23,24,63^, we further determined RNAPII chromatin binding levels. In wild type, the localization of Rpb1, the catalytic subunit of RNAPII, was limited at the centromeres as compared to its level at *adl1* (Fig. 5b, RNAPII (Rpb1)). *ago1∆* increased RNAPII levels at dg and dh to the level comparable to that at *adl1*. Note that *ago1∆* did not increase RNAPII levels at imr3 significantly (*P* = 0.29), suggesting that intrinsic transcriptional activity of imr3 is low^64^. *cid14∆* did not significantly change RNAPII levels in *ago1∆* cells (*P* ≥ 0.24), suggesting that Ago1 acts downstream of H3K9me2 to reduce RNAPII localization at the centromeres. In contrast to *cid14∆*, *mlo3∆* reduced RNAPII levels at dg and dh, consistent with previous reports^25^. Epe1 is a putative H3K9 demethylase that antagonizes heterochromatin assembly^62,65^. Loss of Epe1 restored H3K9me2 and reduced RNAPII chromatin occupancy and GCR rate in *ago1∆* cells (Supplementary Fig. 5). Whereas *cid14∆*, *mlo3∆*, and *epe1∆* restored H3K9me2, only *mlo3∆* and *epe1∆* reduced RNAPII occupancy and GCRs in *ago1∆* cells. These results show that repression of RNAPII might be required for GCR suppression.

### Loss of Mlo3 reduces GCRs in the absence of H3K9 methylation

**Fig. 6:**
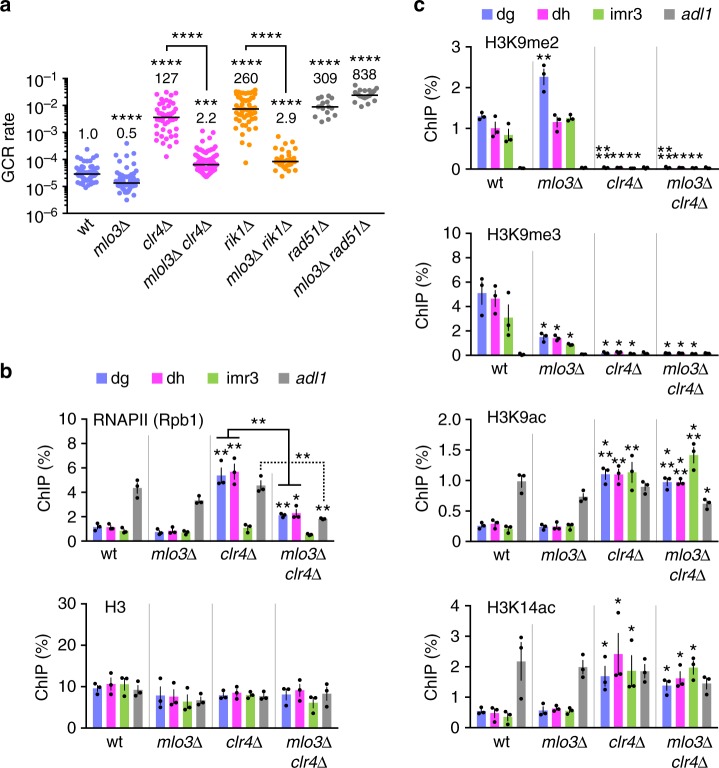
Repression of RNAPII suppresses centromeric gross chromosomal rearrangements (GCRs) in the absence of H3K9 methylation. **a** GCR rates of wild-type, *mlo3∆*, *clr4∆*, *mlo3∆ clr4∆*, *rik1∆*, *mlo3∆ rik1∆*, *rad51∆*, and *mlo3∆ rad51∆* strains (TNF5676, 5764, 5702, 5824, 6121, 6378, 6244, and 6383, respectively). The two-tailed Mann-Whitney test. ***P < 0.001, ****P < 0.0001. **b** Chromatin immunoprecipitation (ChIP) analysis of RNAPII (Rpb1) and H3 in wild-type, *mlo3∆, clr4∆*, and *mlo3∆ clr4∆* strains (TNF5921, 5923, 5948, and 5925, respectively). The two-tailed Student’s *t*-test. *P < 0.05, **P < 0.01. **c** ChIP analysis of H3K9me2, H3K9me3, H3K9ac, and H3K14ac in wild-type, *mlo3∆, clr4∆*, and *mlo3∆ clr4∆* strains. *mlo3∆* reduced the level of H3K9me3 but not that of H3K9me2, suggesting that Mlo3 is required for the transition from H3K9me2 to H3K9me3 state

To examine whether repression of RNAPII suppresses GCRs even in the absence of H3K9 methylation, we deleted *mlo3* in the *clr4∆* mutant and found that *mlo3∆* greatly reduced GCR rate in *clr4∆* cells (Fig. 6a, magenta dots). It has been shown that *mlo3∆* restores chromatin binding of Rik1 in *ago1∆* cells^25^. However, *mlo3∆* suppressed GCRs independently of Rik1, as *mlo3∆* also reduced GCRs of *rik1∆* cells. The homologous recombination factor Rad51 is required to suppress isochromosome formation, but it is not essential for transcription silencing at centromeres^29,53^. Contrary to *clr4∆* cells, *mlo3∆* did not reduce GCR rate in *rad51∆* cells (Fig. 6a, gray dots; Supplementary Fig. 6), showing that *mlo3∆* specifically affects GCRs that occur in heterochromatin-deficient cells. ChIP experiments showed that *clr4∆*, like *ago1∆*, increased RNAPII but not H3 levels at dg and dh, and that *mlo3∆* reduced RNAPII levels in *clr4∆* cells at dg, dh, and *adl1* sites (Fig. 6b). As expected, *mlo3∆* did not restore H3K9me2 and H3K9me3 in *clr4∆* cells (Fig. 6c). Repression of histone acetylation is another feature of heterochromatin^51^. Sir2, Clr3, and Clr6 catalyze deacetylation of histones at different sites, including H3K9 and H3K14, and are involved in transcriptional silencing at the centromeres^66–68^. We found that they are also required for GCR suppression (Supplementary Fig. 7). However, like H3K9 methylation, *mlo3∆* did not significantly change H3K9 and H3K14 acetylation levels (Fig. 6c, *P* ≥ 0.24 and ≥ 0.33, respectively, for H3K9ac and H3K14ac), suggesting that Mlo3 directly affects chromatin binding of RNAPII. These results suggest that the CLRC complex suppresses centromeric GCRs by repressing RNAPII.

### RNAPII and Tfs1/TFIIS cause GCRs in the absence of Clr4

**Fig. 7:**
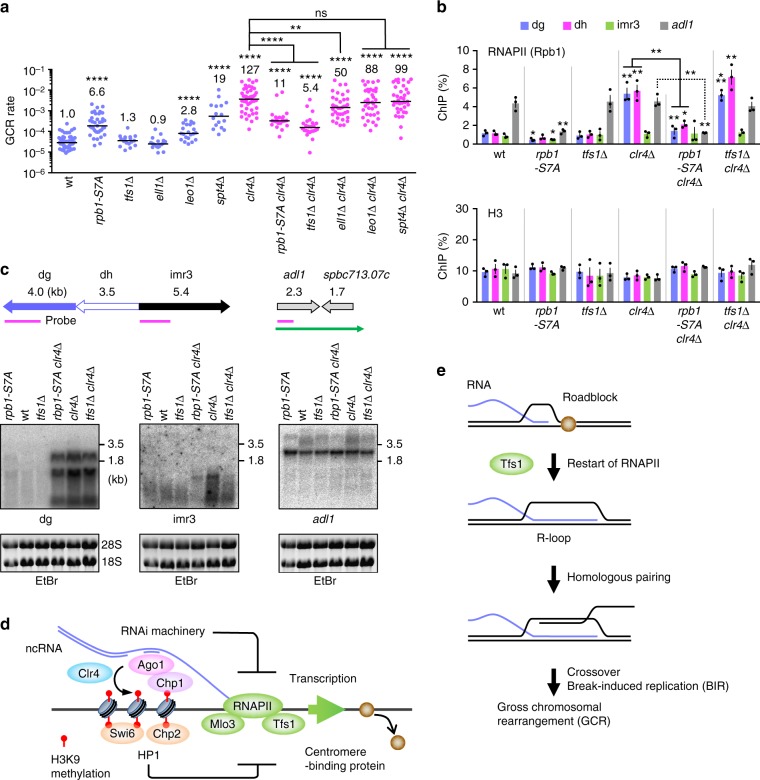
Clr4 suppresses centromeric gross chromosomal rearrangements (GCRs) by repressing transcription that is dependent on RNAPII CTD Ser7 and Tfs1/TFIIS. **a** GCR rates of wild-type, *rpb1-S7A*, *tfs1∆*, *ell1∆*, *leo1∆*, *spt4∆*, *clr4∆*, *rpb1-S7A clr4∆*, *tfs1∆ clr4∆*, *ell1∆ clr4∆*, *leo1∆ clr4∆*, and *spt4∆ clr4∆* strains (TNF5676, 6848, 6688, 7042, 7130, 7055, 5702, 6850, 6726, 7063, 7154, and 7057, respectively). The two-tailed Mann-Whitney test. **P < 0.01, ****P < 0.0001; ns, not significant. **b** Chromatin immunoprecipitation (ChIP) analysis of RNAPII (Rpb1) and H3 in wild-type, *rpb1-S7A*, *tfs1∆*, *clr4∆*, *rpb1-S7A clr4∆*, and *tfs1∆ clr4∆* strains (TNF5921, 6862, 6722, 5948, 6864, and 6799, respectively). The two-tailed Student’s *t*-test. *P < 0.05, ***P < 0.001. **c** Northern blotting using total RNAs prepared from log phase cultures of *rpb1-S7A*, wild-type, *tfs1∆*, *rpb1-S7A clr4∆*, *clr4∆*, and *tfs1∆ clr4∆* strains. Illustrated are the positions of DNA probes used in Northern blotting (magenta bars) and the readthrough transcript of *adl1* (a green arrow). RNAs were separated by 1.0% agarose gel under denatured condition, stained with ethidium bromide (EtBr) (the bottom panel), transferred onto a nylon membrane, and hybridized with specific probes (the top panel). Uncropped images of depicted gels and blots are shown in Supplementary Fig. 12. **d** A model that explains how heterochromatin suppresses GCRs at centromeres. With the aid of the RNAi system, Clr4 catalyzes H3K9 methylation at centromeres. H3K9 methylation marks are recognized by the chromodomain proteins including Clr4, Swi6, Chp2, and Chp1. Both HP1 homologs, Swi6 and Chp2, and an RNAi component Chp1 are required for full suppression of GCRs. In addition to the Clr4 recruitment, RNAi machinery may prevent transcription of noncoding RNAs from centromere repeats to suppress GCRs. RNAPII transcription that depends on CTD Ser7, Mlo3, and Tfs1/TFIIS causes centromeric GCRs possibly by removing DNA binding proteins, such as replication factors, from DNA. **e** Tfs1/TFIIS-dependent transcription might remove the roadblock that binds to DNA and produce R-loops, which facilitate interaction between centromere repeats at non-allelic positions and cause crossover and/or break-induced replication (BIR) that leads to GCRs

Rpb1 CTD consists of YSPTSPS heptapeptide repeats^69^. Ser7 of the CTD is required for transcription of noncoding small nuclear RNAs in human cells^70^. Changing all the Ser7 to Ala, *rpb1-S7A*, reduces chromatin-bound RNAs and H3K9me2 levels at fission yeast centromeres^71,72^. To obtain the direct evidence that RNAPII is involved in centromeric GCRs, we created the *rpb1-S7A* strain that harbored ChL. Consistent with low levels of H3K9me2^72^ (Supplementary Fig. 8a), *rpb1-S7A* slightly increased GCR rate in otherwise wild-type background (Fig. 7a, blue dots). However, *rpb1-S7A* greatly reduced the GCR rate in *clr4∆* cells (Fig. 7a, magenta dots), showing that RNAPII is involved in centromeric GCRs that occur in *clr4∆* cells. Whereas *rpb1-S7A* did not restore H3K9 methylation in *clr4∆* cells (Supplementary Fig. 8a), *rpb1-S7A* reduced Rpb1 localization at centromere repeats and at *adl1* and *act1* genes either in the presence or absence of Clr4 (Fig. 7b and Supplementary Fig. 8b). *rpb1-S7A* also reduced chromatin binding of Rpb3 another subunit of RNAPII^73^ (Supplementary Fig. 8c), suggesting that RNAPII CTD Ser7 is required for chromatin binding of the RNAPII complex. Because levels of RNAPII chromatin binding do not always reflect levels of transcription, we detected noncoding RNAs transcribed from centromere repeats. Northern blotting using total RNAs prepared from yeast cells showed that *clr4∆* increased the amounts of dg, dh, and (less prominently) imr3 RNAs (Fig. 7c and Supplementary Fig. 9a). *rpb1-S7A* slightly increased dg and dh RNAs in otherwise wild-type background, as expected^72^. However, in *clr4∆* cells, *rpb1-S7A* reduced the levels of centromeric noncoding RNAs most prominently at imr3 where transcription levels are low. These results show that RNAPII CTD Ser7 is required for a subset of transcription events in *clr4∆* cells. We detected *adl1* RNAs of ~2.5 and ~5 kb: the long RNAs were the readthrough transcripts that encompassed the downstream converging gene *spbc713.07c*^74^. Interestingly, *rpb1-S7A* specifically reduced the long RNAs of *adl1*. Re-hybridization of the membrane showed that *rpb1-S7A* did not affect the transcription of the *act1* gene that has no converging genes nearby (Supplementary Fig. 9a). These results suggest that RNAPII CTD Ser7 is required for a specific type of transcription that causes centromeric GCRs in the *clr4∆* mutant.

To gain insights into how transcription causes GCRs in the absence of Clr4, we disrupted the genes encoding transcription factors Tfs1, Ell1, Leo1, and Spt4^75^. Among them, Tfs1/TFIIS is the only factor that has been shown to facilitate restart of transcription elongation by trimming 3′-ends of nascent RNAs when RNAPII is paused and backtracked on template DNA^41,42^. In *clr4*^+^ cells, *spt4∆* increased GCR rate (Fig. 7a, blue dots), probably because *spt4∆* impairs transcriptional silencing^76^. In *clr4∆* cells, only *tfs1∆* greatly reduced GCR rate (Fig. 7a, magenta dots). In contrast to *rpb1-S7A*, *tfs1∆* did not significantly change RNAPII levels (Fig. 7b, *P* ≥ 0.22), indicating that RNAPII chromatin binding per se does not cause GCR events. However, *tfs1∆* slightly reduced the levels of centromeric noncoding RNAs in *clr4∆* cells (Fig. 7c and Supplementary Fig. 9a). Interestingly, *tfs1∆* also reduced *adl1* readthrough transcripts, suggesting that Tfs1 facilitates transcription passing through termination sites. *tfs1∆* did not reduce GCRs in *rad51∆* cells (Supplementary Fig. 9b), showing that *tfs1∆* specifically suppresses GCRs that occur in heterochromatin-deficient cells. These results suggest that a specific type of transcription elongation that depends on Tfs1/TFIIS causes GCRs at the centromeres.

## Discussion

Here, we found that heterochromatin suppresses GCRs mediated by centromere repeats. Deletion of Clr4 or Rik1 strongly increased spontaneous formation of isochromosomes whose breakpoints were located in centromere repeats. Mutations in either the SET domain of Clr4 or H3K9 increased GCR rate, suggesting that Clr4 suppresses centromeric GCRs through H3K9 methylation. HP1 homologs Swi6 and Chp2 and the RNAi component Chp1 were the chromodomain proteins essential for full suppression of GCRs. *mlo3∆* and *rpb1-S7A* impaired chromatin binding of RNAPII and reduced GCRs in the *clr4∆* mutant, showing that Clr4-dependent H3K9 methylation suppresses GCRs by repressing RNAPII. Strikingly, deletion of Tfs1/TFIIS, which facilitates restart of paused and backtracked RNAPII, greatly reduced GCRs in the *clr4∆* mutant without changing RNAPII chromatin binding levels. These results suggest that heterochromatin suppresses the Tfs1-dependent transcription that leads to GCRs between centromere repeats.

Chromodomain proteins are the readers of the H3K9 methylation mark. Among them, Swi6 is required for stable binding of cohesin complexes at the centromeres, and it also facilitates early replication of the centromeres by recruiting Dbf4/Dfp1-dependent kinase (DDK)^31,77,78^. However, *swi6∆* did not increase GCR rate, indicating that neither cohesin enrichment nor replication timing control at the centromeres is essential to suppress GCRs. In contrast to each single mutation, the *swi6∆ chp2∆* double mutation increased GCR rate, indicating that the redundant function of Swi6 and Chp2 such as inhibition of RNAPII localization at the centromeres^10,56^ is important for GCR suppression. A previous report has also shown that *swi6∆* does not alter GCR rate but increases the proportion of isochromosomes among GCR products^38^. Similar to the case with GCRs, meiotic recombination at the centromeres is increased only when both Swi6 and Chp2 are eliminated^40^. Nonetheless, HP1 homologs are not the only chromodomain proteins required to suppress GCRs, as GCR rate of *swi6∆ chp2∆* was not as high as that of *clr4∆* cells. *swi6∆ chp2∆* and *chp1∆* synergistically increased GCR rate to the level similar to that of *clr4∆*, suggesting that both HP1 and the RNAi machinery are required for the full suppression of GCRs (Fig. 7d).

The RITS complex recruits the CLRC complex and facilitates H3K9 methylation at the centromeres. Loss of Clr4 eliminates H3K9 di-methylation and tri-methylation and increases RNAPII localization at centromere repeats^8,58^. Like *clr4∆*, *ago1∆* reduced H3K9 methylation and increased RNAPII localization, resulting in high incidence of isochromosome formation. However, GCR rate of *ago1∆* was higher than that of *clr4∆*, showing that Ago1 has a Clr4-independent function in GCR suppression. A discrepancy between RNAi mutants (*ago1∆*, *dcr1∆*, and *rdp1∆*) and *clr4∆* phenotypes has been observed in the formation of uniparental disomy (UPD), a pair of homologous chromosomes originating from only one parent^79^. However, in contrast to the case of UPD, Ago1 may have a unique function to suppress GCRs, which is independent of other RNAi factors including Dcr1, Rdp1, Chp1, Tas3, Arb1, and Arb2. Human Ago1 directly binds to RNAPII^80^, and Drosophila Ago2 interacts with the negative elongation factor NELF and represses heat-shock genes under normal conditions^81^. Thus, Ago1 might directly affect transcription and suppress GCRs. This study provides the evidence that transcriptional repression is important to suppress GCRs between centromere repeats. Mlo3 as well as RNAPII localizes to the centromeres in *clr4∆* cells^8,24^, and Yra1/Mlo3 directly binds to the CTD of Rpb1 in budding yeast^23^. *mlo3∆* reduced chromatin binding of RNAPII and bypassed the requirement of Clr4 methyltransferase to suppress GCRs at the centromeres. In addition, mutations in the largest subunit of RNAPII, *rpb1-S7A*, also reduced RNAPII chromatin binding and bypassed the requirement of Clr4 for GCR suppression. Transcriptional repression by heterochromatin seems to be important to suppress homology-mediated GCRs not only at the centromeres but also at other chromosomal loci, e.g., subtelomeres^82^.

How does Tfs1/TFIIS-dependent transcription cause GCRs that are mediated by centromere repeats? During transcription, DNA supercoils are formed in front of and behind RNAPII, hybridization of RNA to the template DNA creates R-loops, nucleosomes are disassembled and reassembled, and proteins are detached from template DNA. RNAPII is paused and backtracked when it encounters DNA-binding proteins^83,84^. After backtracking, Tfs1/TFIIS cleaves nascent RNAs by enhancing the intrinsic nuclease activity of RNAPII and facilitates the restart of RNAPII^41,42^. Here, we found that *tfs1∆* greatly reduced GCRs in *clr4∆* cells. In contrast to GCRs, *tfs1∆* did not significantly change the level of RNAPII chromatin binding and only slightly reduced the amount of centromeric noncoding RNAs, showing that neither RNAPII binding nor transcription per se induces GCRs. Interestingly, the effect of *tfs1∆* on transcription levels was most prominent at imr3 repeats where the intrinsic transcription activity is low. Tfs1 facilitates passing through the transcription termination site and produces readthrough transcripts of the *adl1* gene. We propose a model in which, with the aid of Tfs1, RNAPII competes with DNA-binding proteins that block transcription elongation (Fig. 7e). Interestingly, *clr4∆* impairs the centromere localization of the Smc5-Smc6 complex that promotes a conservative way of homologous recombination^85^. Thus, the removal of DNA-binding proteins such as Smc5-Smc6 can cause illegitimate recombination. Tfs1-dependent transcription may also block the progression of replication forks, as the replication fork protection machinery is important to suppress recombination at centromeres in the heterochromatin mutant^38^. Rad52 recombinase binds to the centromeres during S phase in an RNAi mutant, and Rad51 recombinase is essential in RNAi mutants^39^. Drosophila lacking Suv39 histone methyltransferase accumulates spontaneous DNA damage in heterochromatin^86^. Dnmt3a and Dnmt3b that catalyze DNA methylation, another epigenetic mark of heterochromatin in mammalian cells, suppress recombination at the centromeres^87^. However, our previous study has shown that *clr4∆* increases recombination between inverted repeats in the centromeres by ~2-fold^54^, arguing against the notion that *clr4∆* merely increases DNA lesions that cause GCRs. Clr4 may affect the choice of recombination pathways at the centromeres. Either crossover or break-induced replication (BIR) between centromere inverted repeats results in isochromosome formation^53,88^. Rad51 suppresses isochromosome formation by promoting non-crossover recombination at the centromeres^53^. In the absence of Rad51, GCRs occur in a manner-dependent on the crossover-specific endonuclease Mus81^53^, demonstrating that crossover is the mechanism of GCRs in *rad51∆* cells. Tfs1-dependent transcription passing through pausing or termination sites may extend the length of RNA:DNA hybrids and produce R-loops (Fig. 7e). In mammals, R-loops are sometimes formed at transcription termination sites, and BRCA1 recombinase binds to DNA damage derived from R-loops^89^. A recent paper showed that R-loops induce BIR in budding yeast^90^. R-loops that contain single-stranded DNA may facilitate the pairing between homologous sequences and initiate BIR (Fig. 7e). Further studies are required to address how Tfs1-dependent transcription causes GCRs between centromere repeats. We also found that *tfs1∆*, *mlo3∆*, and *rpb1-S7A* reduced chromosome loss in the *clr4∆* background (Supplementary Fig. 9c), suggesting that transcriptional repression is important not only for GCR suppression but also for correct segregation of chromosomes. Unlike *rpb1-S7A* and *mlo3∆*, *tfs1∆* reduced hypersensitivity to the microtubule-destabilizing drug, thiabendazole (TBZ) of *clr4∆* cells (Supplementary Fig. 9d), making Tfs1/TFIIS-dependent transcription a critical target of heterochromatin to maintain the integrity and the function of the centromeres.

It is believed that heterochromatin assembles on the centromeres to ensure faithful segregation of chromosomes^31,32,91^. However, heterochromatin is not always formed at the centromeres. In fission yeast strain CBS2777 and pathogenic fungus *Candida lusitaniae*, no heterochromatin or transcriptional silencing was observed at the centromeres that were devoid of repeat sequences^33,34^. In chicken DT40 cells, heterochromatin is assembled at the repetitive centromeres but not at the non-repetitive centromeres^35^. Together with these links between heterochromatin and DNA repeats, our studies suggest that one of the important roles of centromeric heterochromatin is to suppress GCRs that are mediated by centromere repeats. Interestingly, de-repression of repetitive sequences including centromeric satellite DNA is observed in some kinds of cancer cells^6^. During the process of aging, heterochromatin is globally lost and frequencies of genome alterations increase^92–94^. We propose that heterochromatin represses transcription of noncoding repeats in the genome to prevent GCRs between the repetitive sequences.

## Methods

### Strains and media

Fission yeast strains used in this study are listed in Supplementary Table 1. Yeast cells were grown at 30 °C in YE, EMM, YNB, and 5FOA media supplemented with appropriate amino acids at a final concentration of 225 mg L^−1^ as described previously^54^. YNB medium contained 1.7 g L^−1^ of yeast nitrogen base (BD Biosciences, Difco 233520), 5 g L^−1^ of ammonium sulfate (Nacalai Tesque, 02619-15), and 2% glucose. YNB medium was supplemented with 1 g L^−1^ of 5-fluoroorotic acid (Apollo Scientific, PC4054) and 56 mg L^−1^ of uracil to make 5FOA medium. Solid media contained 1.5% agarose (Nacalai Tesque, 01028-85). Yeast transformation was performed by the lithium acetate method. The transformants that contain the kanamycin, hygromycin, or nourseothricin resistance gene were selected on the media supplemented with G418 (Nacalai Tesque, 09380-86), hygromycin B (Nacalai Tesque, 09287-84), or clonNAT (Werner BioAgents, 96736-11-7) at a final concentration of 100 µg mL^−1^. *clr4-R406A,N409A,H410A* (*clr4-set*), *mlo3K165A,K167A* (*mlo3KA*), and *mlo3K165R,K167R* (*mlo3KR*) mutant strains were created by the pop-in/pop-out gene replacement^95^: pTN1220 plasmid containing the wild-type *ura4*^+^ and mutant *clr4-set* genes was digested with NgoMIV and introduced into *ura4-D18* mutant cells. pTN1179 containing *ura4*^+^ and *mlo3KA* and pTN1178 containing *ura4*^+^ and *mlo3KR* were digested with HpaI and introduced into *ura4-D18* cells. Ura^+^ transformants were selected on EMM plates, and then, Ura^–^ progenies resulting from *ura4*^+^ pop-out were selected on 5FOA plates. We performed PCR and DNA sequencing to confirm correct integration of the *clr4* and *mlo3* mutations.

### Plasmids

A 1.8 kb HindIII–SspI fragment containing the *ura4*^+^ gene was introduced between HindIII–EcoRV sites of pBluescript II KS^+^ (Stratagene) to make pTN782. *clr4-set*, *mlo3KA*, and *mlo3KR* mutant genes were constructed by a two-step PCR method. From yeast genomic DNA, a 0.7 kb PCR fragment was produced using clr4-1 and clr4-NHR-F primers, and a 1.0 kb fragment using clr4-NHR-R and clr4-2 primers, independently. These partially overlapping PCR fragments were mixed and used for the 2nd PCR in the presence of clr4-1 and clr4-2 primers. A 1.4 kb SpeI–PvuII restriction fragment prepared from the 2nd PCR product was introduced between SpeI–NaeI sites of pTN782 to make pTN1220. A 2.0 kb genomic region that contains the *mlo3*^+^ gene was amplified using mlo3-1 and mlo3-5, and digested with XbaI at one site. A 1.9 kb restriction fragment with XbaI–blunt ends was introduced between XbaI–NaeI sites of pTN782 to make pTN1169. From yeast genomic DNA, a 1.0 kb PCR fragment was produced using mlo3-1 and mlo3-KA-R primers, and a 0.7 kb fragment using mlo3-KA-F and mlo3-4 primers. These partially overlapping PCR fragments were mixed and used for the 2nd PCR in the presence of mlo3-1 and mlo3-4 primers. A 1.0 kb SacI–XbaI restriction fragment of the 2nd PCR product that contains the *mlo3KA* mutation was introduced between SacI–XbaI sites of pTN1169 to make pTN1179. mlo3-KR-R and mlo3-KR-F primers were used in place of mlo3-KA-R and mlo3-KA-F to make pTN1178 that contains the *mlo3KR* mutation. A 9.6 kb XbaI–EcoRI fragment containing cen1 sequence from pRS140^96^ was introduced between XbaI–EcoRI sites of pUC19 to make pTN834. From yeast genomic DNA, a 1.7 kb region that contains a portion of dh was amplified using dh-1 and dh-2 primers. A 1.5 kb NheI–ClaI restriction fragment of the PCR product was introduced between SpeI–ClaI sites of pBluescript II KS^+^ to make pTN770. A 2.3 kb region that contains a portion of imr3 was amplified using otr3-2 and imr3-XhoI-R primers. A 1.7 kb PvuII–MfeI restriction fragment of the PCR product was introduced between HincII–EcoRI sites of pBluescript II KS^+^ to make pTN1226. A 0.9 kb region that contains a portion of the *adl1* gene was amplified using adl1-F and adl1-R primers. A 0.9 kb XbaI–ApaI restriction fragment of the PCR product was introduced between XbaI–ApaI sites of pBluescript II KS^+^ to make pTN1227. A 2.1 kb region that contains a portion of the *act1* gene was amplified using act1-F and act1-R primers. A 1.9 kb XhoI–EcoRV restriction fragment of the PCR product was introduced between XhoI–EcoRV sites of pBluescript II KS^+^ to make pTN1225. DNA sequencing confirmed that no mutations were introduced during PCR amplification.

### Gross chromosomal rearrangement (GCR) assay

Rates of spontaneous GCR were determined by the fluctuation analysis. Yeast cells were incubated for 6–9 days on EMM + UA plates, and 10 mL of EMM + UA medium was inoculated with a single colony formed on the EMM + UA plates. After 2-days incubation, cells were plated onto YNB + UA and 5FOA + A media. After incubation for 6–12 days, the number of colonies formed on YNB + UA and 5FOA + A plates were counted to determine the number of Leu^+^ and that of Leu^+^ Ura^–^ cells, respectively. Leu^+^ Ura^–^ colonies formed on 5FOA + A plates were incubated on EMM + UA plates and then replicated onto EMM + A and EMM + U plates to confirm Ura^–^ and to inspect Ade^+/–^, respectively. The number of Leu^+^ Ura^–^ Ade^–^ cells indicative of GCR was obtained by subtracting the number of Leu^+^ Ura^–^ Ade^+^ cells from that of Leu^+^ Ura^–^ cells. Using the number of Leu^+^ cells and that of Leu^+^ Ura^–^ Ade^–^ cells in 10 mL of EMM + UA culture, we determined GCR rate by means of the fluctuation analysis^44^.

### Pulse field gel electrophoresis (PFGE)

From parental and GCR (Leu^+^ Ura^–^ Ade^–^) clones obtained from biologically independent experiments, chromosomal DNAs were prepared in 1.6% low melting agarose plugs (Nacalai Tesque, 01161-12) as described previously^54^. Chromosomal DNAs were separated in 0.55% Certified Megabase agarose gel (Bio-Rad, 161-3109) using CHEF-DRII system (Bio-Rad) under the following conditions. Broad-range PFGE: 1500 s pulse time at 2 V cm^−1^ for 42 h and then, 180 s pulse time at 2.4 V cm^−1^ for 4 h, at 4 °C in 1× TAE buffer (40 mM Tris-acetate, 1 mM EDTA). Short-range PFGE: from 40 to 70 s pulse time at 4.2 V cm^−1^ for 24 h, at 4 °C in 0.5× TBE buffer (89 mM Tris-borate, 2 mM EDTA). After electrophoresis, DNAs were stained with 0.2 µg mL^−1^ of EtBr (Nacalai Tesque, 14631-94) and detected using a Typhoon FLA9000 (GE Healthcare).

### Southern blotting

After EtBr staining, agarose gel was irradiated with 300 mJ ultraviolet (UV) light using GS Gene Linker (Bio-Rad) for DNA fragmentation, and then soaked into 800 mL of alkaline buffer (1.2 M NaCl, 0.4 M NaOH) for 40 min with gentle shaking to denature DNA. DNA was transferred to ClearTrans nylon membrane 0.45 µm (Wako, 039-22673) by capillary action in 25 mM sodium phosphate buffer (pH 6.5) and covalently attached to the membrane by 150 mJ UV irradiation. A 0.6 kb EcoRI–EcoRI fragment prepared from pTN755^29^, α-^32^P-dCTP (PerkinElmer Life Sciences, NEG013H), and Random primer labeling kit ver. 2 (Takara, 6045) were used to prepare radioactive probe A. Radioactive signals were detected using BAS2500 phosphorimager (Fuji Film).

### PCR analysis of GCR products

After separation of chromosomal DNA by PFGE, GCR products were recovered from agarose gel using a FastGene Gel/PCR Extraction kit (Nippon Genetics, FG-91302). KOD FX Neo polymerase (Toyobo, KFX-201) was utilized to amplify cnt3–imr3 junctions, whereas Q5 polymerase (New England Biolabs, M0491) was used to amplify irc3. PCR products were separated by 1.7% Seakem GTG agarose gel (Lonza, 50070) electrophoresis in 1× TBE buffer, stained with 0.2 µg mL^−1^ of EtBr, and detected using a Typhoon FLA9000. PCR primers used in this assay are listed in Supplementary Table 2.

### Chromatin immunoprecipitation (ChIP)

ChIP was performed as described previously^54^. 1.5 × 10^8^ cells from log-phase cultures in YE media supplemented with leucine, uracil, adenine, and histidine (YE4S) were collected by centrifugation, and suspended in 60 mL of EMM. After the addition of formaldehyde (Sigma-Aldrich, F8775) to a final concentration of 1%, the cell suspension was vigorously mixed for 15 min at room temperature. The cell suspension was further mixed for 5 min, after the addition of 3 mL of 2.5 M glycine to neutralize the crosslinker. Mouse antibodies against H3K9me2^97^, H3K9me3^97^, H3K9ac^98^, H3K14ac^98^, Rpb1 (Millipore, CTD4H8, 05-623), and FLAG (Sigma-Aldrich, F1804), and rabbit antibodies against histone H3 (Abcam, ab1791) were used. Mouse and rabbit antibodies were attached to Dynabeads M-280 sheep anti-Mouse IgG (Invitrogen, 11202D) and Dynabeads M-280 sheep anti-Rabbit IgG (Invitrogen, 11204D), respectively. DNAs in whole-cell extracts and immunoprecipitates were quantified by real-time PCR using SYBR FAST (Thermo Fisher, 4385614) in a StepOnePlus real-time PCR system (Applied Biosystems). The primers used in ChIP are listed in Supplementary Table 3.

### Northern blotting

Northern blotting was carried out as described previously^53^. From 1 × 10^9^ log-phase cells grown in YE4S media, RNA was extracted by heating and freezing cells in the presence of phenol and SDS. 10 µg of total RNAs was suspended in 8.5 µL of MOPS buffer (20 mM MOPS pH 7.0, 2 mM NaAc, 1 mM EDTA) supplemented with 8% formaldehyde, 50% deionized formamide, and 10 µg mL^−1^ EtBr, and heat denatured by incubation at 55 °C for 60 min. RNAs were separated by 1.0% PrimeGel agarose LE (TaKaRa, 5801 A) gel/2.2 M formaldehyde electrophoresis in MOPS buffer. RNAs stained with EtBr were detected using a Typhoon FLA9000. After soaking the gel in 50 mM NaOH (Nacalai Tesque, 31511-05) for 20 min, RNAs were transferred to a ClearTrans nylon membrane 0.45 µm by capillary action in alkaline transfer buffer (10 mM NaOH, 3 M NaCl) and covalently attached to the membrane by 150 mJ UV irradiation. A 2.0 kb KpnI–KpnI fragment prepared from pTN834, a 0.9 kb NsiI–XbaI fragment from pTN770, a 1.7 kb PstI–XhoI fragment from pTN1226, a 0.9 kb XbaI–ApaI fragment from pTN1227, and a 1.9 kb XhoI–EcoRV fragment from pTN1225, were used to prepare radioactive DNA probes for the detection of dg, dh, imr3, *adl1*, and *act1* RNAs, respectively. Radioactive signals were detected using a BAS2500 phosphorimager.

### Statistical analysis

The two-tailed Mann–Whitney test and the two-tailed Fisher’s exact test were performed using GraphPad Prism version 6.0 g for Mac (GraphPad Software). The two-tailed Student’s *t*-test was performed using Excel (Microsoft).

## Supplementary information

Supplementary Information

## Data Availability

The datasets generated during the current study are available in the Dryad repository^99^. The plasmids created in the study can be obtained from National Bio Resource Project (NBRP) in Japan.
