# Supplementary material for: Heterochromatin suppresses gross chromosomal rearrangements at centromeres by repressing Tfs1/TFIIS-dependent transcription
Source: Commun Biol. 2019 Jan 11;2:17. doi: 10.1038/s42003-018-0251-z (PMC6329695; doi:10.1038/s42003-018-0251-z)
Supplement: Supplementary file 1 — Supplementary Information [file 42003_2018_251_MOESM1_ESM.pdf]

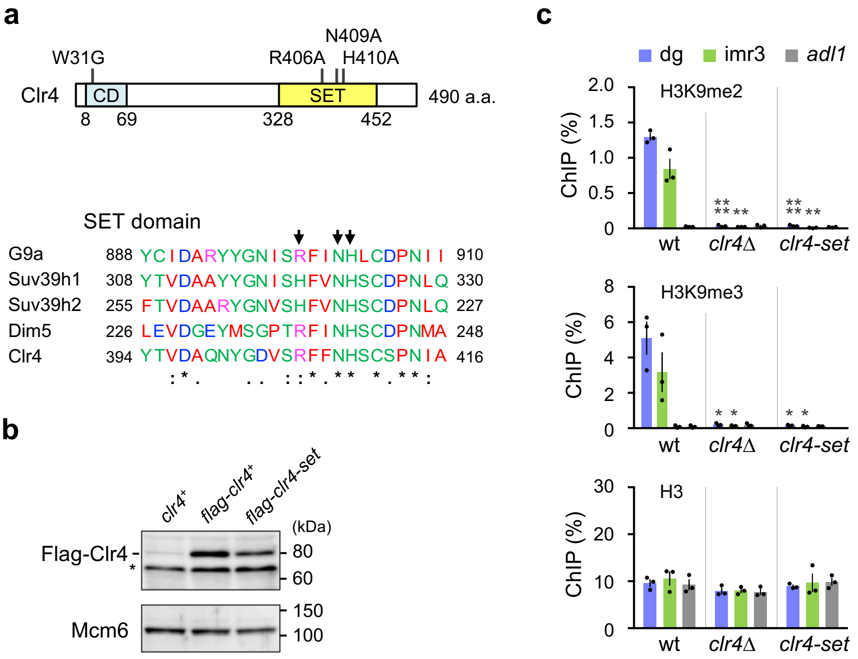

Supplementary Fig. 1 The mutation in the SET domain of Clr4, *clr4-set*, eliminates H3K9 methylation at centromeres. (**a**) The Clr4 protein has the chromodomain (CD) and the SET domain. Positions of *clr4-W31G* and *clr4-set* mutations are indicated. Sequence alignment of a portion of the SET domains of *Homo sapiens* G9a, Suv39h1, and Suv39h2, *Neurospora crassa* Dim5, and *Schizosaccharomyces pombe* Clr4, prepared using Clustal Omega[1]. The residues altered in the *clr4-set* mutant (R406, N409, and H410) are indicated by arrows. (**b**) Immunostaining of Flag-Clr4. Cell extracts were prepared from *clr4⁺*, *flag-clr4⁺*, and *flag-clr4-set* cells (TNF2605, 5981, and 6280, respectively). Mcm6 was detected as a loading control. An asterisk indicates a non-specific band. Uncropped images of depicted gels and blots are shown in Supplementary Fig. 13. (**c**) Chromatin immunoprecipitation (ChIP) analysis was performed to determine the level of H3K9me2, H3K9me3, and H3 at centromere repeats (dg and imr3) and at a non-centromeric region of chr2 (*adl1)* in wild-type, *clr4Δ*, and *clr4-set* strains (TNF5921, 5948, and 6169, respectively). DNA levels were quantified by real time PCR, and percentages of input DNA were obtained. Data are presented as the mean ± s.e.m. from three biologically independent experiments. Dots represent individual mesurements from distinct samples. Statistical significance of differences relative to wild type was determined using the two-tailed Student's *t*-test. * $P < 0.05$, ** $P < 0.01$, **** $P < 0.0001$.

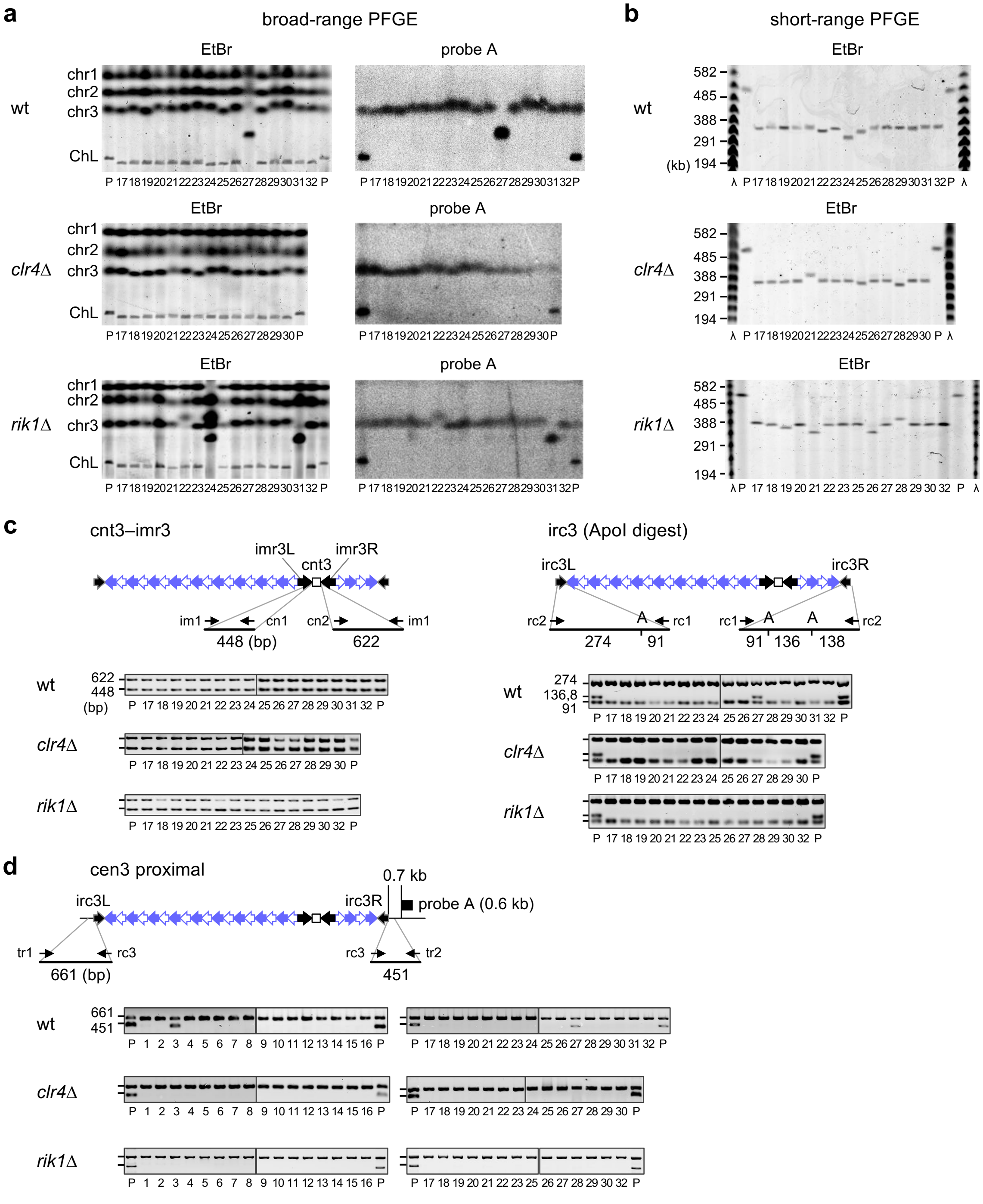

Supplementary Fig. 2 Clr4 and Rik1 suppress the isochromosome formation whose breakpoints are located in centromere repeats. (**a**) Southern blot analysis of gross chromosomal rearrangement (GCR) products formed in wild-type, *clr4Δ,* and *rik1Δ* strains (TNF5676, 5702, and 6121, respectively). Chromosomal DNAs of parental and independent GCR clones were separated by broad-range pulse field gel electrophoresis (PFGE) and stained with ethidium bromide (EtBr). Positions of chr1, chr2, chr3, and ChL (5.7, 4.6, ~3.5, and 0.5 Mb, respectively) in the parental strain are indicated on the left of the panel. DNAs were transferred onto a nylon membrane and hybridized with probe A. P, Parental. (**b**) Chromosomal DNAs were separated by short-range PFGE and stained with EtBr. Sizes of the λ DNA ladder are indicated on the left of the panel. (**c**) Breakpoints were determined by PCR reactions using GCR products recovered from agarose gel. Both sides of cnt3–imr3 junctions were amplified in the reaction containing im1, cn1, and cn2 primers. irc3L and irc3R were amplified using rc1 and rc2 primers, and the PCR products were digested by ApoI and separated by agarose gel electrophoresis. A, ApoI. (**d**) Both sides of cen3 proximal regions were amplified from the GCR products of wild type and *clr4Δ*. Uncropped images of depicted gels and blots are shown in Supplementary Figs. 14 and 15.

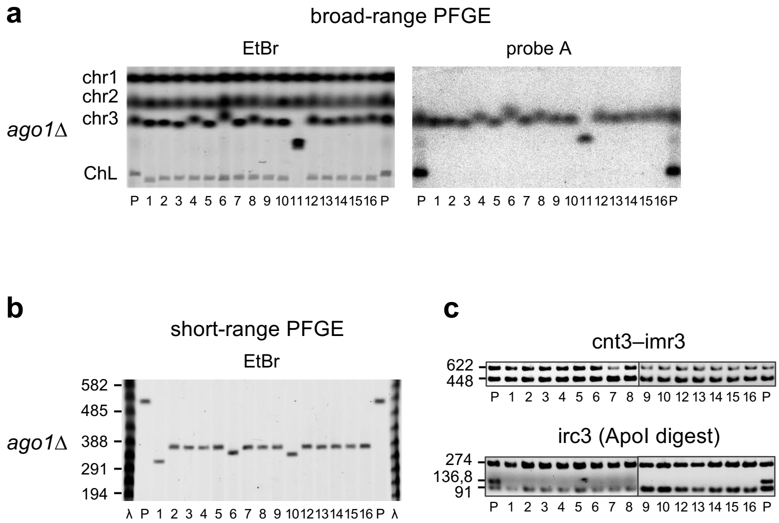

Supplementary Fig. 3 Ago1 suppresses the isochromosome formation whose breakpoints are located in centromere repeats. (**a**) Gross chromosomal rearrangement (GCR) products formed in *ago1Δ* cells (TNF5688). Chromosomal DNAs were separated by broad-range pulse field gel electrophoresis (PFGE) and stained with ethidium bromide (EtBr). DNAs transferred onto a nylon membrane were hybridized with probe A. P, Parental. (**b**) Chromosomal DNAs were separated by short-range PFGE and stained with EtBr. Sizes of the λ DNA ladder are indicated on the left of the panel. (**c**) Breakpoints were determined as described in Supplementary Fig. 2c. Uncropped images of depicted gels and blots are shown in Supplementary Fig. 16.

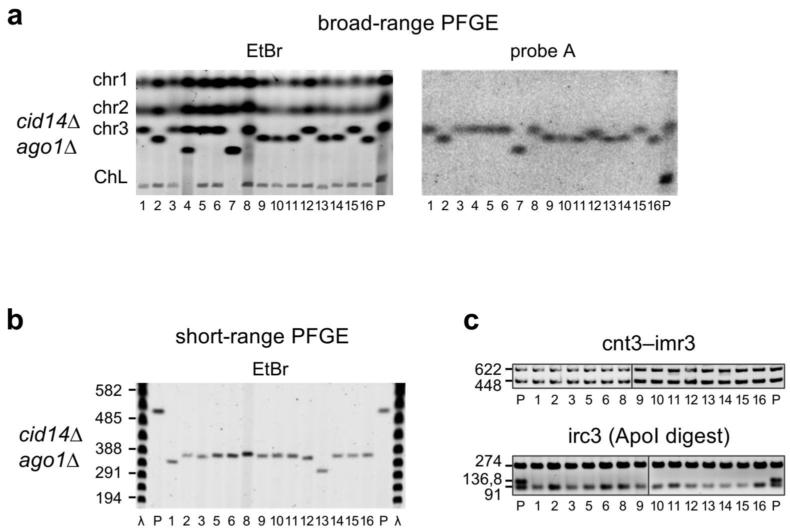

Supplementary Fig. 4 The isochromosome formation in *cid14Δ ago1Δ* cells. (**a**) Gross chromosomal rearrangement (GCR) products formed in *cid14Δ ago1Δ* strains (TNF6411). Chromosomal DNAs were separated by broad-range pulse field gel electrophoresis (PFGE) and stained with ethidium bromide (EtBr). DNAs transferred onto a nylon membrane were hybridized with probe A. P, Parental. (**b**) Chromosomal DNAs were separated by short-range PFGE and stained with EtBr. Sizes of the λ DNA ladder are indicated on the left of the panel. (**c**) Breakpoints were determined as described in Supplementary Fig. 2c. Uncropped images of depicted gels and blots are shown in Supplementary Fig. 17.

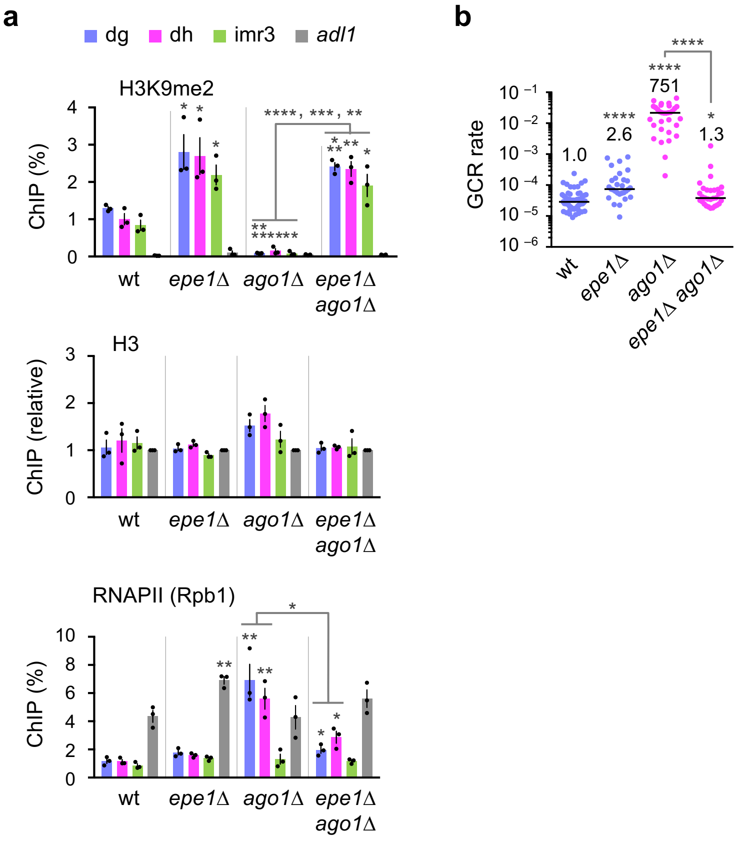

Supplementary Fig. 5 ***epe1Δ*** **suppresses gross chromosomal rearrangements (GCRs) in** ***ago1Δ*** **cells.** (**a**) Chromatin immunoprecipitation (ChIP) analysis of H3K9me2, H3, and RNAPII (Rpb1) in wild-type, *epe1Δ*, *ago1Δ*, and *epe1Δ ago1Δ* strains (TNF5921, 7349, 5922, and 7343, respectively). The value relative to that of *adl1* is shown in the case of H3. Data are presented as the mean ± s.e.m. from three biologically independent experiments. The two-tailed Student's *t*-test, * $P < 0.05$, ** $P < 0.01$, *** $P < 0.001$, **** $P < 0.0001$. (**b**) GCR rates of wild-type, *epe1Δ*, *ago1Δ*, and *epe1Δ ago1Δ*, strains (TNF5676, 6109, 5688, and 7325, respectively). Each dot represents the GCR rate determined using a single colony formed on EMM+UA plates in scatter plots. Lines represent the median. The GCR rate relative to that of wild type is indicated on the top of each column. The two-tailed Mann-Whitney test.

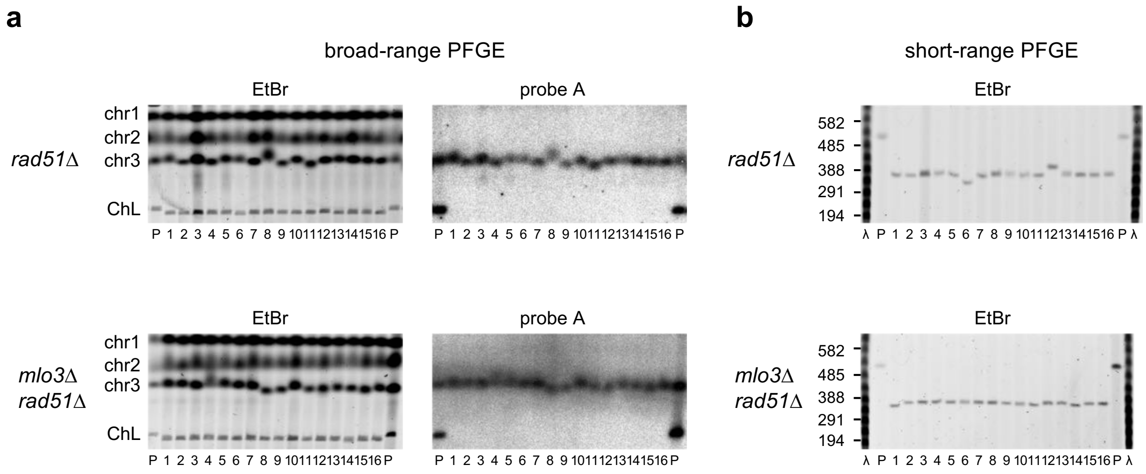

Supplementary Fig. 6 The isochromosome formation in *mlo3Δ rad51Δ* cells. (**a**) Gross chromosomal rearrangement (GCR) products formed in *rad51Δ* and *mlo3Δ rad51Δ* strains (TNF6244 and 6383, respectively). Chromosomal DNAs were separated by broad-range pulse field gel electrophoresis (PFGE) and stained with ethidium bromide (EtBr). DNAs transferred onto a nylon membrane were hybridized with probe A. P, Parental. (**b**) Chromosomal DNAs were separated by short-range PFGE and stained with EtBr. Sizes of the λ DNA ladder are indicated on the left of the panel. Uncropped images of depicted gels and blots are shown in Supplementary Fig. 18.

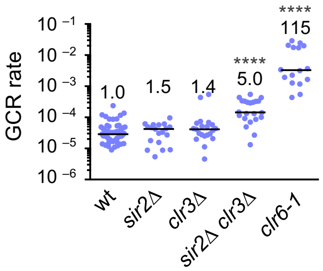

Supplementary Fig. 7 Histone deacetylases Sir2, Clr3, and Clr6 are required for GCR suppression. GCR rates of wild-type, *sir2Δ*, *clr3Δ*, *sir2Δ clr3Δ*, and *clr6-1* strains (TNF5676, 7341, 7359, 7357, and 7345, respectively). The two-tailed Mann-Whitney test, **** $P < 0.0001$.

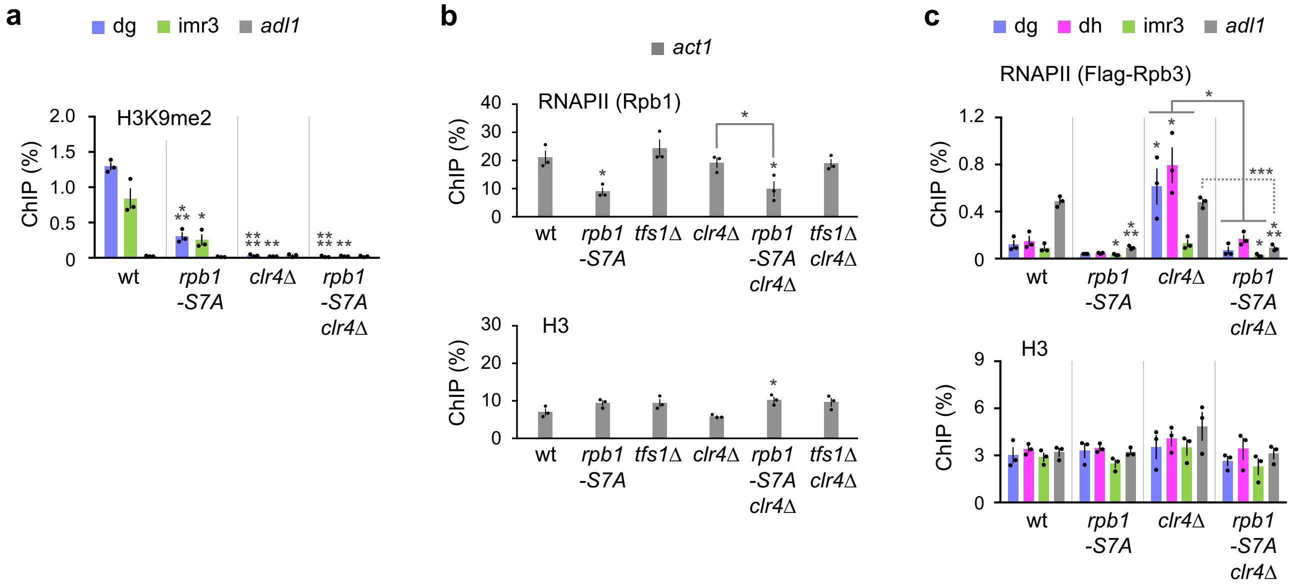

Supplementary Fig. 8 *rpb1-S7A* reduces chromatin binding of RNAPII. (**a**) Chromatin immunoprecipitation (ChIP) analysis of H3K9me2 in wild-type, *rpb1-S7A*, *clr4Δ*, and *rpb1-S7A clr4Δ* strains (TNF5921, 6862, 5948, and 6864, respectively). Data are presented as the mean ± s.e.m. from three biologically independent experiments. The two-tailed Student's *t*-test, * $P < 0.05$, ** $P < 0.01$, *** $P < 0.001$, **** $P < 0.0001$. (**b**) ChIP analysis of RNAPII (Rpb1) and H3 in wild-type, *rpb1-S7A*, *tfs1Δ*, *clr4Δ*, *rpb1-S7A clr4Δ*, and *tfs1Δ clr4Δ* strains at the highly transcribed *act1* gene (TNF5921, 6862, 6722, 5948, 6864, and 6799, respectively). (**c**) ChIP analysis of RNAPII (Flag-Rpb3) and H3 in wild-type, *rpb1-S7A, clr4Δ*, and *rpb1-S7A clr4Δ* strains (TNF6931, 6943, 6933, and 6945, respectively).

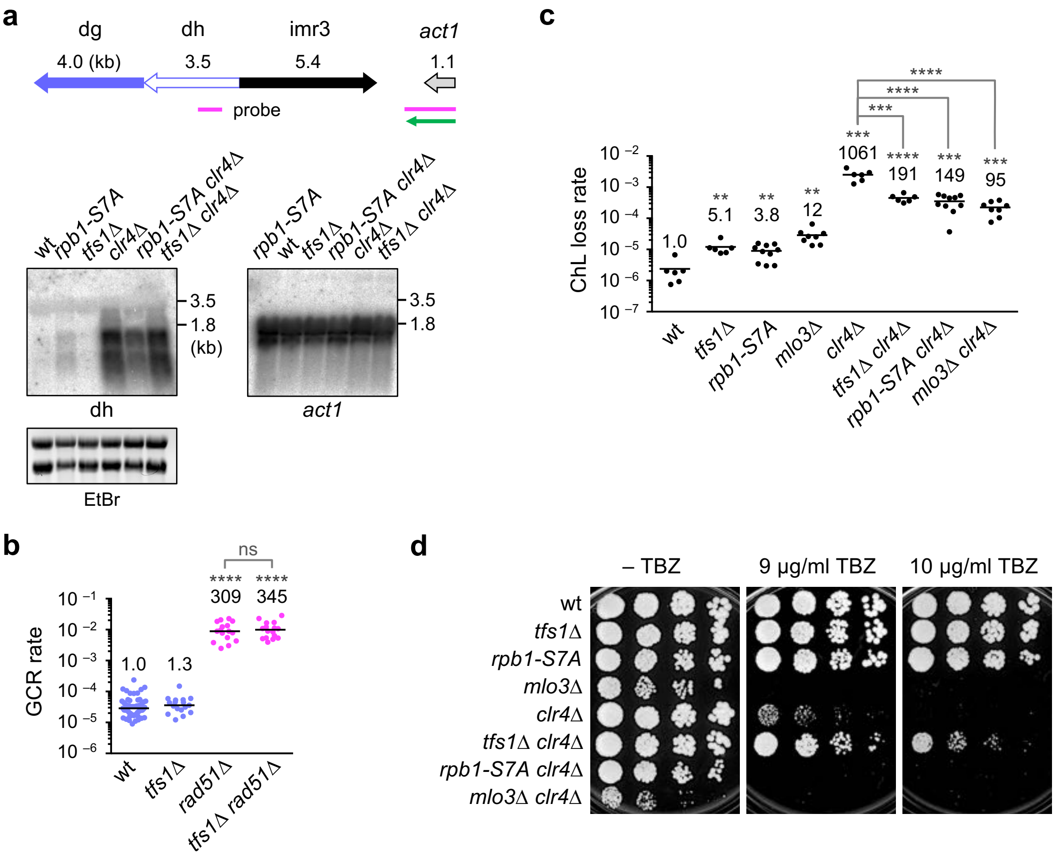

Supplementary Fig. 9 Effects of *rpb1-S7A, tfs1Δ,* or *mlo3Δ* on transcription and centromere functions. (**a**) Northern blotting using total RNAs of wild-type, *rpb1-S7A*, *tfs1Δ*, *clr4Δ*, *rpb1-S7A clr4Δ*, and *tfs1Δ clr4Δ* strains (TNF5921, 6862, 6722, 5948, 6864, and 6799, respectively). Illustrated are the positions of DNA probes used in Northern blotting (magenta bars) and the *act1* transcribed region (a green arrow). RNAs were separated by 1.0% agarose gel under denatured condition, stained with ethidium bromide (EtBr) (the bottom panel), transferred onto a nylon membrane, and hybridized with specific probes (the top panel). The membrane that has been hybridized with the *adl1* probe (Fig. 7c) was re-hybridized with the *act1* probe. Uncropped images of depicted gels and blots are shown in Supplementary Fig. 19. (**b**) Gross chromosomal rearrangement (GCR) rates of wild-type, *tfs1Δ*, *rad51Δ*, and *tfs1Δ rad51Δ* strains (TNF5676, 6688, 6244, and 7163, respectively). The two-tailed Mann-Whitney test, **** $P < 0.0001$; ns, not significant. (**c**) Rates of the ChL loss in wild-type, *tfs1Δ*, *rpb1-S7A*, *mlo3Δ*, *clr4Δ*, *tfs1Δ clr4Δ*, *rpb1-S7A clr4Δ*, and *mlo3Δ clr4Δ* strains (TNF5676, 6688, 6848, 5764, 5702, 6726, 6850, and 5824, respectively). Each dot represents the ChL loss rate determined using a single colony formed on EMM plates in scatter plots. Lines represent the mean. The ChL loss rate relative to that of the wild-type strain is indicated on the top of each column. The two-tailed Student's *t*-test, ** $P < 0.01$, *** $P < 0.001$. (**d**) Log-phase cultures of wild-type, *tfs1Δ, rpb1-S7A, mlo3Δ, clr4Δ*, *tfs1Δ clr4Δ, rpb1-S7A clr4Δ,* and *mlo3Δ clr4Δ* strains (TNF5921, 6722, 6862, 5923, 5948, 6799, 6864, and 5925, respectively) were 5-fold serially diluted with distilled water and spotted onto YE+LUA supplemented with thiabendazole (TBZ) at a final concentration of 9 and 10 μg mL^{-1}.

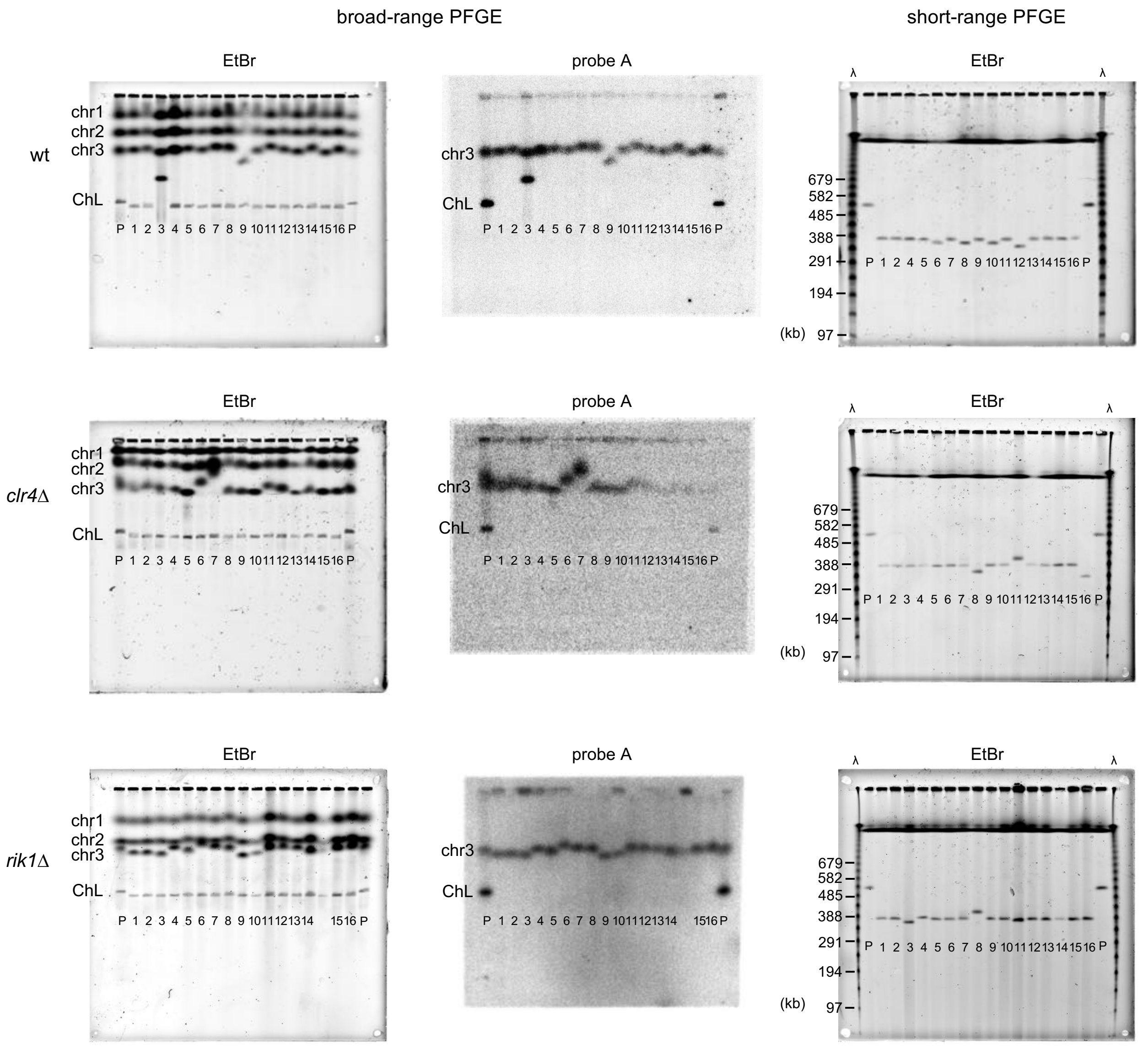

Supplementary Fig. 10 Full-sized scans of gels and blots in Figs. 2c and 2d.

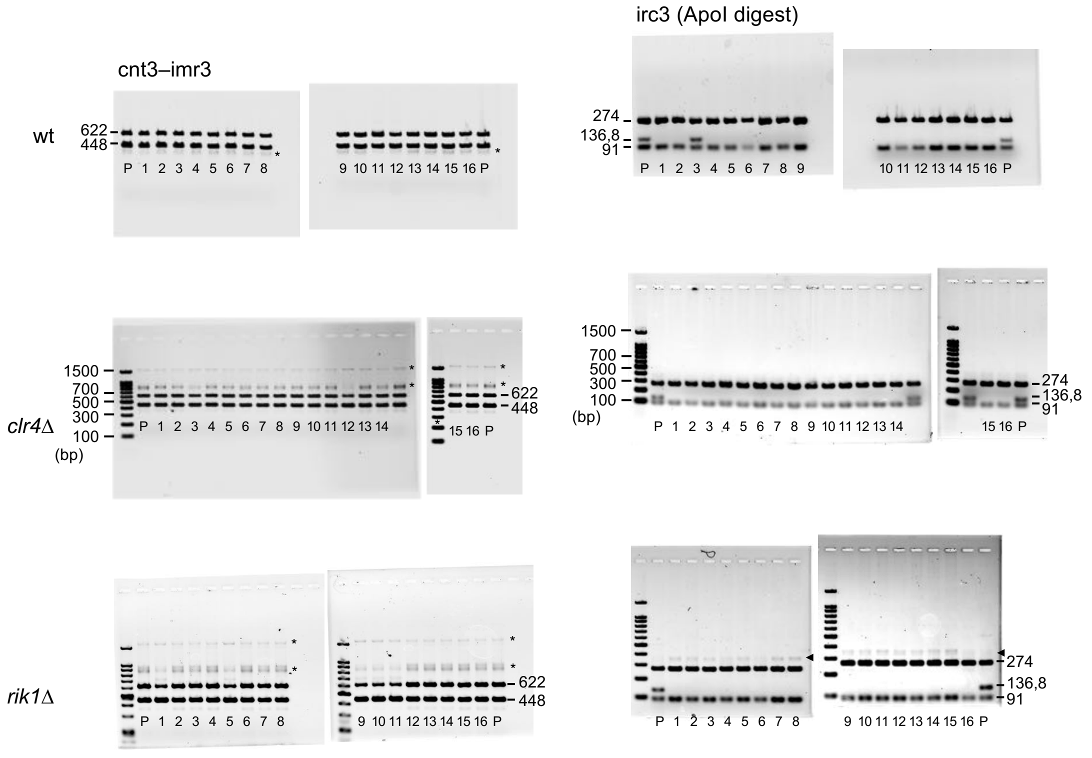

Supplementary Fig. 11 Full-sized scans of gels in Fig. 2f. Asterisks represent nonspecific bands. Arrowheads show uncompleted digested PCR products.

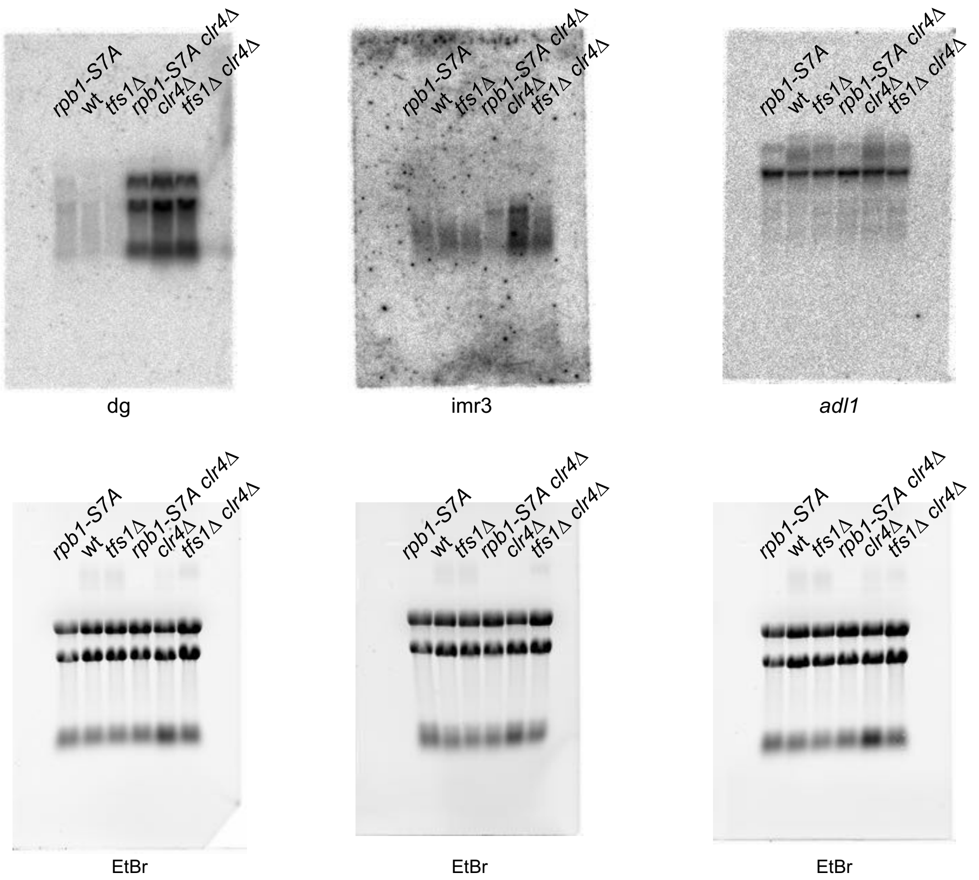

Supplementary Fig. 12 Full-sized scans of gels and blots in Fig. 7c.

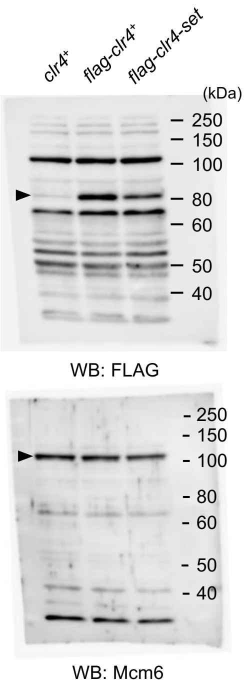

Supplementary Fig. 13 Full-sized scans of blots in Supplementary Fig. 1b.

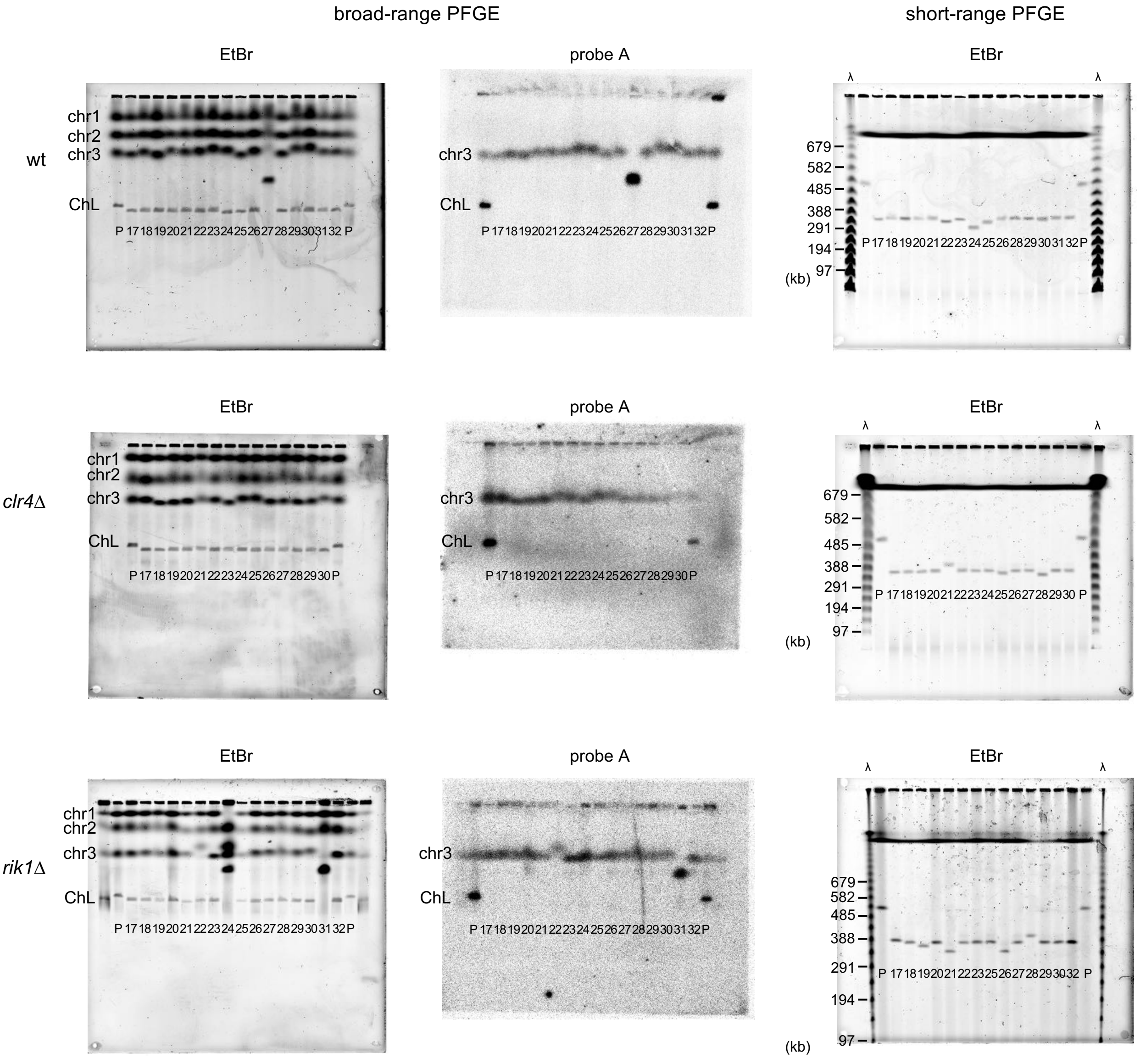

Supplementary Fig. 14 Full-sized scans of gels and blots in Supplementary Figs. 2a and 2b.

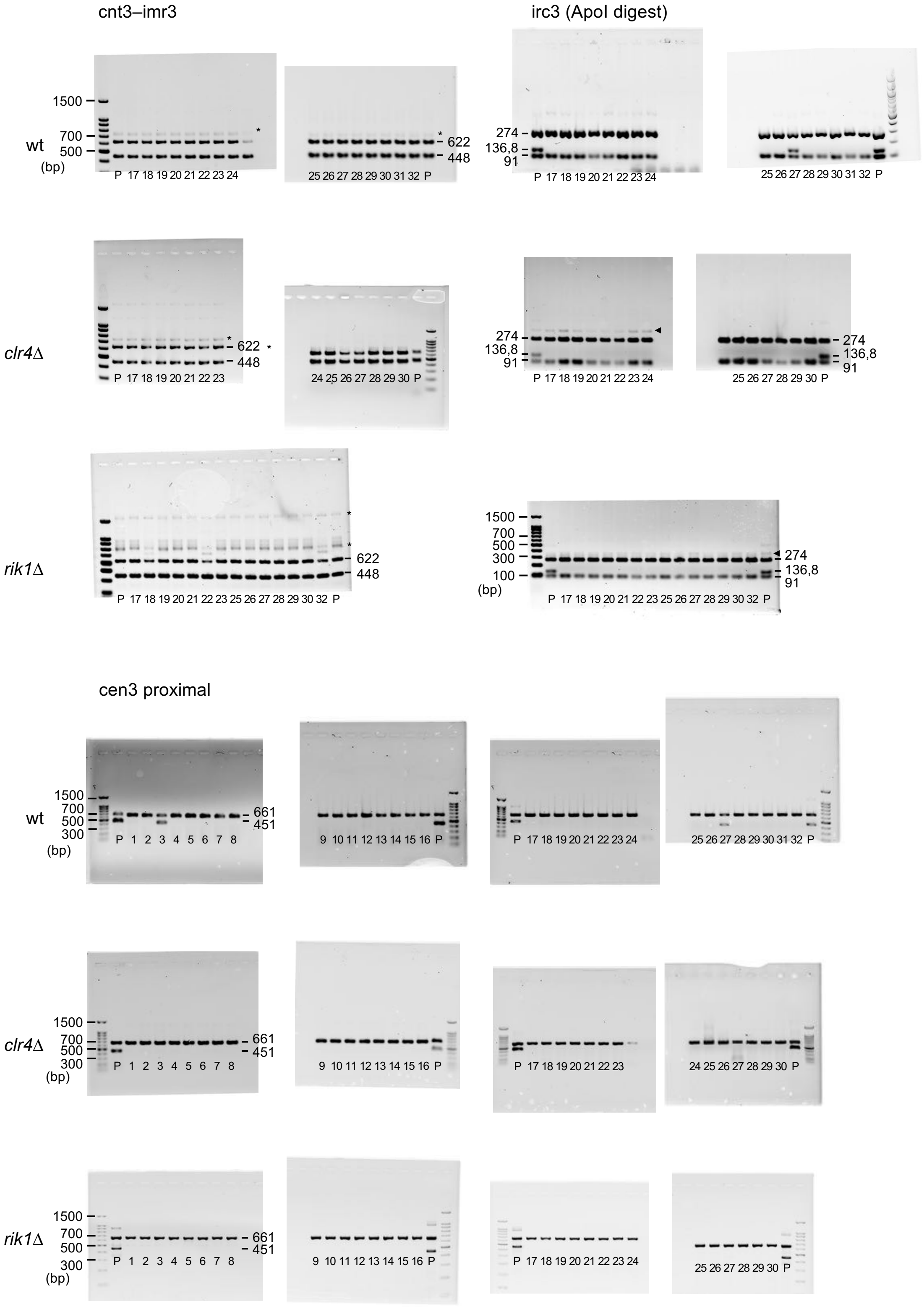

Supplementary Fig. 15 Full-sized scans of gels in Supplementary Figs. 2c and 2d. Asterisks represent nonspecific bands. Arrowheads show uncompleted digested PCR products.

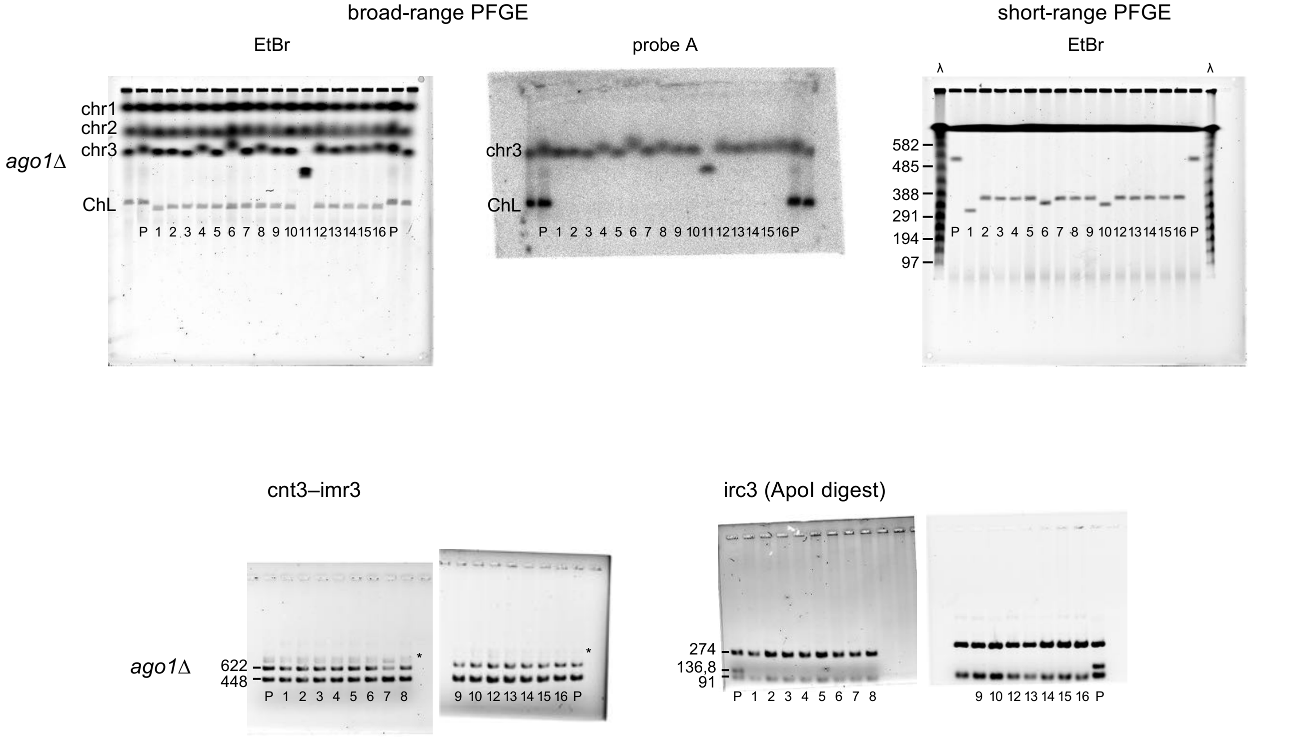

Supplementary Fig. 16 Full-sized scans of gels and blots in Supplementary Fig. 3. Asterisks represent nonspecific bands.

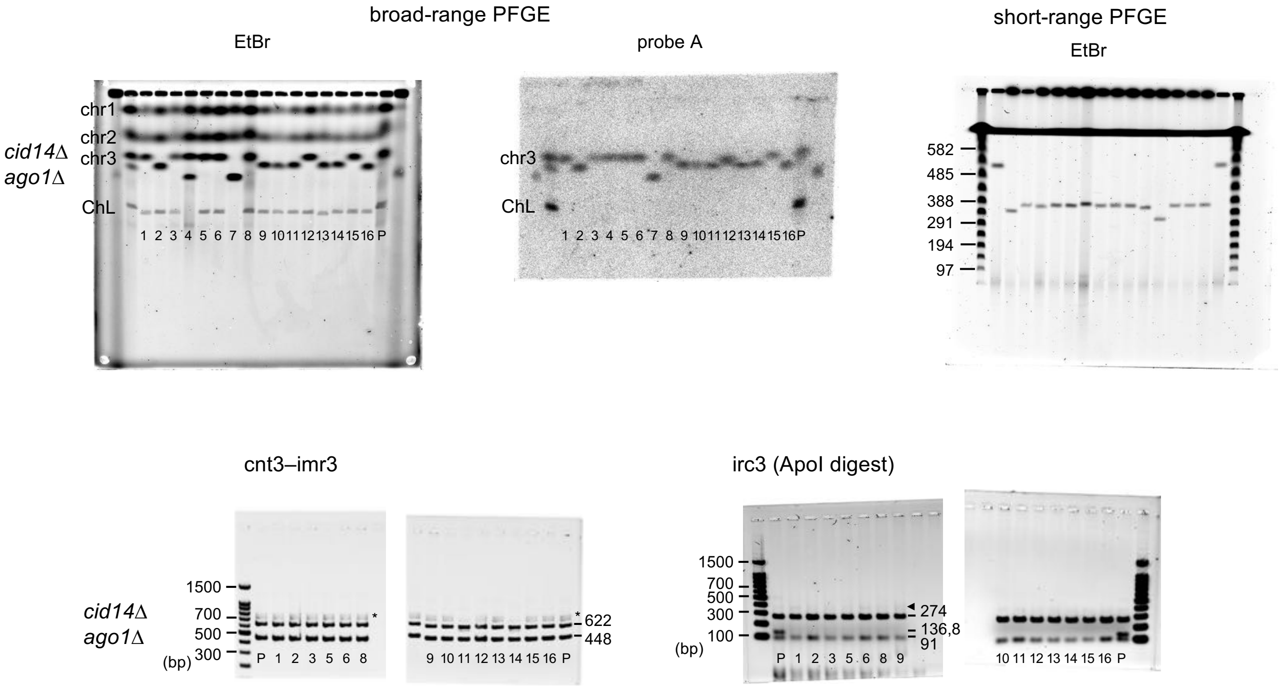

Supplementary Fig. 17 Full-sized scans of gels and blots in Supplementary Fig. 4. Asterisks represent nonspecific bands. Arrowheads show uncompleted digested PCR products.

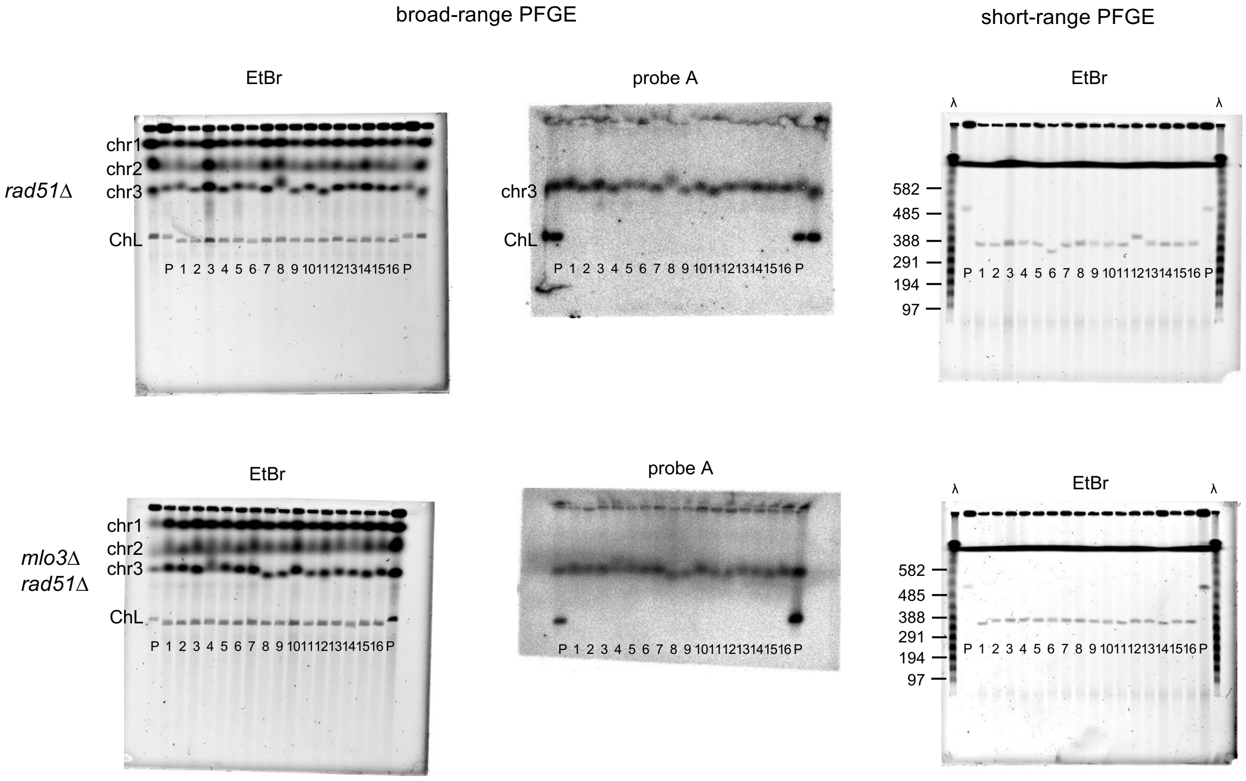

Supplementary Fig. 18 Full-sized scans of gels and blots in Supplementary Fig. 6.

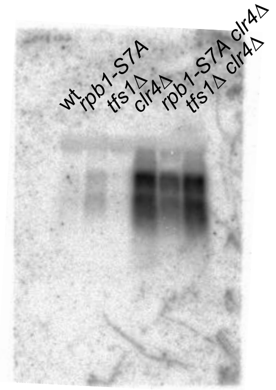

dg

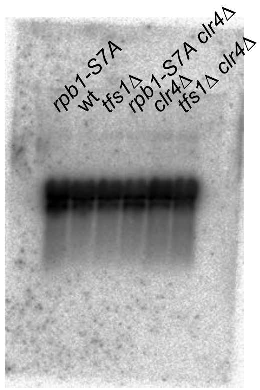

act1

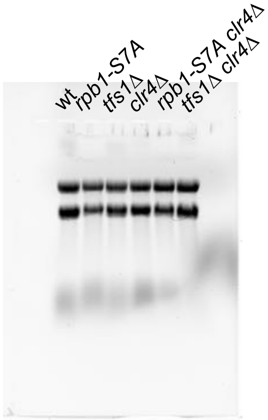

EtBr

Supplementary Fig. 19 Full-sized scans of gels and blots in Supplementary Fig. 9a.

Supplementary Table 1. The yeast strains used in this study.

strain	genotype
TNF3896	h^-, *smt0*, *ade6Δ-D*, *ura4-D18*, *leu1-32*, ChL
TNF5440	h^-, *smt0*, *ade6Δ-D*, *ura4-D18*, *leu1-32*, ChL, *clr4::kanMX6*
TNF5676	h^-, *smt0*, *mat2-3::natMX6*, *ade6Δ-D*, *ura4-D18*, *leu1-32*, ChL
TNF5702	h^-, *smt0*, *mat2-3::natMX6*, *ade6Δ-D*, *ura4-D18*, *leu1-32*, ChL, *clr4::hphMX6*
TNF5701	h^-, *smt0*, *mat2-3::natMX6*, *ade6Δ-D*, *ura4-D18*, *leu1-32*, ChL, *rec12::hphMX6*
TNF5766	h^-, *smt0*, *mat2-3::natMX6*, *ade6Δ-D*, *ura4-D18*, *leu1-32*, ChL, *rec12::hphMX6*, *clr4::kanMX6*
TNF6121	h^-, *smt0*, *mat2-3::natMX6*, *ade6Δ-D*, *ura4-D18*, *leu1-32*, ChL, *rik1::kanMX6*
TNF6958	h^-, *smt0*, *mat2-3::natMX6*, *ade6Δ-D*, *ura4-D18*, *leu1-32*, ChL, *clr4-R406A*,*N409A*,*H410A*
TNF6155	h^-, *smt0*, *mat2-3::natMX6*, *ade6Δ-D*, *ura4-D18*, *leu1-32*, ChL, *mlo3-K165A*,*K167A*
TNF6157	h^-, *smt0*, *mat2-3::natMX6*, *ade6Δ-D*, *ura4-D18*, *leu1-32*, ChL, *mlo3-K165R*,*K167R*
TNF5738	h^-, *smt0*, *mat2-3::natMX6*, *ade6Δ-D*, *ura4-D18*, *leu1-32*, ChL, *h3.1/h4.1::*$his3^+$, *h3.3/h4.3::*$arg3^+$
TNF6223	h^-, *smt0*, *mat2-3::natMX6*, *ade6Δ-D*, *ura4-D18*, *leu1-32*, ChL, *h3.1/h4.1::*$his3^+$, *h3.3/h4.3::*$arg3^+$, *h3.2-K9A*
TNF5802	h^-, *smt0*, *mat2-3::natMX6*, *ade6Δ-D*, *ura4-D18*, *leu1-32*, ChL, *h3.1/h4.1::*$his3^+$, *h3.3/h4.3::*$arg3^+$, *h3.2-K9R*
TNF6012	h^-, *smt0*, *mat2-3::natMX6*, *ade6Δ-D*, *ura4-D18*, *leu1-32*, ChL, *clr4-W31G*
TNF5706	h^-, *smt0*, *mat2-3::natMX6*, *ade6Δ-D*, *ura4-D18*, *leu1-32*, ChL, *swi6::hphMX6*
TNF5685	h^-, *smt0*, *mat2-3::natMX6*, *ade6Δ-D*, *ura4-D18*, *leu1-32*, ChL, *chp2::hphMX6*
TNF5900	h^-, *smt0*, *mat2-3::natMX6*, *ade6Δ-D*, *ura4-D18*, *leu1-32*, ChL, *swi6::kanMX6*, *chp2::hphMX6*
TNF5708	h^-, *smt0*, *mat2-3::natMX6*, *ade6Δ-D*, *ura4-D18*, *leu1-32*, ChL, *chp1::hphMX6*
TNF6151	h^-, *smt0*, *mat2-3::natMX6*, *ade6Δ-D*, *ura4-D18*, *leu1-32*, ChL, *swi6::kanMX6*, *chp2::hphMX6*, *chp1::hphMX6*
TNF5689	h^-, *smt0*, *mat2-3::natMX6*, *ade6Δ-D*, *ura4-D18*, *leu1-32*, ChL, *ago1::hphMX6*
TNF7335	h^-, *smt0*, *mat2-3::natMX6*, *ade6Δ-D*, *ura4-D18*, *leu1-32*, ChL, *tas3::kanMX6*
TNF7337	h^-, *smt0*, *mat2-3::natMX6*, *ade6Δ-D*, *ura4-D18*, *leu1-32*, ChL, *arb1::kanMX6*
TNF7331	h^-, *smt0*, *mat2-3::natMX6*, *ade6Δ-D*, *ura4-D18*, *leu1-32*, ChL, *arb2::kanMX6*
TNF7333	h^-, *smt0*, *mat2-3::natMX6*, *ade6Δ-D*, *ura4-D18*, *leu1-32*, ChL, *rdp1::kanMX6*
TNF5687	h^-, *smt0*, *mat2-3::natMX6*, *ade6Δ-D*, *ura4-D18*, *leu1-32*, ChL, *dcr1::hphMX6*

TNF6153	h^{-}, *smt0*, *mat2-3::natMX6*, *ade6Δ-D*, *ura4-D18*, *leu1-32*, ChL, *cid14::kanMX6*
TNF5764	h^{-}, *smt0*, *mat2-3::natMX6*, *ade6Δ-D*, *ura4-D18*, *leu1-32*, ChL, *mlo3::hphMX6*
TNF6411	h^{-}, *smt0*, *mat2-3::natMX6*, *ade6Δ-D*, *ura4-D18*, *leu1-32*, ChL, *cid14::kanMX6*, *ago1::hphMX6*
TNF6188	h^{-}, *smt0*, *mat2-3::natMX6*, *ade6Δ-D*, *ura4-D18*, *leu1-32*, ChL, *mlo3::hphMX6*, *ago1::kanMX6*
TNF5824	h^{-}, *smt0*, *mat2-3::natMX6*, *ade6Δ-D*, *ura4-D18*, *leu1-32*, ChL, *mlo3::kanMX6, clr4::hphMX6*
TNF6378	h^{-}, *smt0*, *mat2-3::natMX6*, *ade6Δ-D*, *ura4-D18*, *leu1-32*, ChL, *mlo3::hphMX6, rik1::kanMX6*
TNF6244	h^{-}, *smt0*, *mat2-3::natMX6*, *ade6Δ-D*, *ura4-D18*, *leu1-32*, ChL, *rad51::kanMX6*
TNF6383	h^{-}, *smt0*, *mat2-3::natMX6*, *ade6Δ-D*, *ura4-D18*, *leu1-32*, ChL, *mlo3::hphMX6*, *rad51::kanMX6*
TNF6848	h^{-}, *smt0*, *mat2-3::natMX6*, *ade6Δ-D*, *ura4-D18*, *leu1-32*, ChL, *rpb1-CTD-S7A:kanMX6*
TNF6688	h^{-}, *smt0*, *mat2-3::natMX6*, *ade6Δ-D*, *ura4-D18*, *leu1-32*, ChL, *tfs1::kanMX6*
TNF7042	h^{-}, *smt0*, *mat2-3::natMX6*, *ade6Δ-D*, *ura4-D18*, *leu1-32*, ChL, *ell1::kanMX6*
TNF7130	h^{-}, *smt0*, *mat2-3::natMX6*, *ade6Δ-D*, *ura4-D18*, *leu1-32*, ChL, *leo1::kanMX6*
TNF7055	h^{-}, *smt0*, *mat2-3::natMX6*, *ade6Δ-D*, *ura4-D18*, *leu1-32*, ChL, *spt4::kanMX6*
TNF6850	h^{-}, *smt0*, *mat2-3::natMX6*, *ade6Δ-D*, *ura4-D18*, *leu1-32*, ChL, *rpb1-CTD-S7A:kanMX6*, *clr4::hphMX6*
TNF6726	h^{-}, *smt0*, *mat2-3::natMX6*, *ade6Δ-D*, *ura4-D18*, *leu1-32*, ChL, *tfs1::kanMX6*, *clr4::hphMX6*
TNF7063	h^{-}, *smt0*, *mat2-3::natMX6*, *ade6Δ-D*, *ura4-D18*, *leu1-32*, ChL, *ell1::kanMX6*, *clr4::hphMX6*
TNF7154	h^{-}, *smt0*, *mat2-3::natMX6*, *ade6Δ-D*, *ura4-D18*, *leu1-32*, ChL, *leo1::kanMX6*, *clr4::hphMX6*
TNF7057	h^{-}, *smt0*, *mat2-3::natMX6*, *ade6Δ-D*, *ura4-D18*, *leu1-32*, ChL, *spt4::kanMX6*, *clr4::hphMX6*
TNF6109	h^{-}, *smt0*, *mat2-3::natMX6*, *ade6Δ-D*, *ura4-D18*, *leu1-32*, ChL, *epe1::hphMX6*
TNF7325	h^{-}, *smt0*, *mat2-3::natMX6*, *ade6Δ-D*, *ura4-D18*, *leu1-32*, ChL, *epe1::hphMX6*, *ago1::hphMX6*
TNF7341	h^{-}, *smt0*, *mat2-3::natMX6*, *ade6Δ-D*, *ura4-D18*, *leu1-32*, ChL, *sir2::hphMX6*
TNF7359	h^{-}, *smt0*, *mat2-3::natMX6*, *ade6Δ-D*, *ura4-D18*, *leu1-32*, ChL, *clr3::hphMX6*
TNF7357	h^{-}, *smt0*, *mat2-3::natMX6*, *ade6Δ-D*, *ura4-D18*, *leu1-32*, ChL, *sir2::hphMX6, clr3::hphMX6*
TNF7345	h^{-}, *smt0*, *mat2-3::natMX6*, *ade6Δ-D*, *ura4-D18*, *leu1-32*, ChL, *clr6-1*

TNF7163	h^{-}, *smt0*, *mat2-3::natMX6*, *ade6Δ-D*, *ura4-D18*, *leu1-32*, ChL, *tfs1::kanMX6*, *rad51::kanMX6*
TNF5921	h^{-}, *smt0*, *mat2-3::natMX6*, *ade6Δ-D*, *ura4-D18*, *leu1-32*
TNF6276	h^{-}, *smt0*, *mat2-3::natMX6*, *ade6Δ-D*, *ura4-D18*, *leu1-32*, *cid14::kanMX6*
TNF5923	h^{-}, *smt0*, *mat2-3::natMX6*, *ade6Δ-D*, *ura4-D18*, *leu1-32*, *mlo3::hphMX6*
TNF5922	h^{-}, *smt0*, *mat2-3::natMX6*, *ade6Δ-D*, *ura4-D18*, *leu1-32*, *ago1::hphMX6*
TNF6550	h^{-}, *smt0*, *mat2-3::natMX6*, *ade6Δ-D*, *ura4-D18*, *leu1-32*, *cid14::kanMX6*, *ago1::hphMX6*
TNF6210	h^{-}, *smt0*, *mat2-3::natMX6*, *ade6Δ-D*, *ura4-D18*, *leu1-32*, *mlo3::hphMX6*, *ago1::kanMX6*
TNF5948	h^{-}, *smt0*, *mat2-3::natMX6*, *ade6Δ-D*, *ura4-D18*, *leu1-32*, *clr4::kanMX6*
TNF5925	h^{-}, *smt0*, *mat2-3::natMX6*, *ade6Δ-D*, *ura4-D18*, *leu1-32*, *clr4::hphMX6*, *mlo3::kanMX6*
TNF6862	h^{-}, *smt0*, *mat2-3::natMX6*, *ade6Δ-D*, *ura4-D18*, *leu1-32*, *rpb1-CTD-S7A:kanMX6*
TNF6722	h^{-}, *smt0*, *mat2-3::natMX6*, *ade6Δ-D*, *ura4-D18*, *leu1-32*, *tfs1::kanMX6*
TNF6864	h^{-}, *smt0*, *mat2-3::natMX6*, *ade6Δ-D*, *ura4-D18*, *leu1-32*, *rpb1-CTD-S7A:kanMX6*, *clr4::hphMX6*
TNF6799	h^{-}, *smt0*, *mat2-3::natMX6*, *ade6Δ-D*, *ura4-D18*, *leu1-32*, *tfs1::kanMX6*, *clr4::hphMX6*
TNF2605	h^{+}, *ura4-D18*
TNF5981	h^{+}, *ura4-D18, 3×flag-clr4*
TNF6280	h^{+}, *ura4-D18, 3×flag-clr4-R406A*,*N409A*,*H410A*
TNF7349	h^{-}, *smt0*, *mat2-3::natMX6*, *ade6Δ-D*, *ura4-D18*, *leu1-32*, *epe1::hphMX6*
TNF7343	h^{-}, *smt0*, *mat2-3::natMX6*, *ade6Δ-D*, *ura4-D18*, *leu1-32*, *epe1::hphMX6*, *ago1::hphMX6*
TNF6169	h^{-}, *smt0*, *mat2-3::natMX6*, *ade6Δ-D*, *ura4-D18*, *leu1-32*, *clr4-R406A*,*N409A*,*H410A*
TNF6931	h^{-}, *smt0*, *mat2-3::natMX6*, *ade6Δ-D*, *ura4-D18*, *leu1-32*, *flag-rpb3*
TNF6943	h^{-}, *smt0*, *mat2-3::natMX6*, *ade6Δ-D*, *ura4-D18*, *leu1-32*, *flag-rpb3*, *rpb1-CTD-S7A:kanMX6*
TNF6933	h^{-}, *smt0*, *mat2-3::natMX6*, *ade6Δ-D*, *ura4-D18*, *leu1-32*, *flag-rpb3*, *clr4::hphMX6*
TNF6945	h^{-}, *smt0*, *mat2-3::natMX6*, *ade6Δ-D*, *ura4-D18*, *leu1-32*, *flag-rpb3*, *rpb1-CTD-S7A:kanMX6*, *clr4::hphMX6*

ChL [*ubc11::LEU2*$^{+}$, *spcc1322.09::ura4*$^{+}$, *ade6*$^{+}$] is a derivative of Ch16.

Supplementary Table 2. The sequence of the primers used in this study.

primer	stock #	sequences
cn1	TN68	5'- AACCGCAACAAACGATTAGC
cn2	TN69	5'- CGGAATTAGAAAGATTGATGATTTG
im1	TN60	5'- AAGTTTTGATGCTCAACAAATGGC
rc1	TN956	5'- CATTAAAAATCAACAAGTCTTGTCC
rc2	TN1772	5'- GTTACTATGGATAAAGATAATTGTTT
rc3	TN2278	5'- CCGTTAGTGAACGTAAATAATGAAACC
tr1	TN2279	5'- ACAAGCGTACTTGACATGCG
tr2	TN2280	5'- GCTTGCAGCTGAAATGTTTATTCG
clr4-1	TN674	5'- AACTCCAACGCCTCGAACAGCTGC
clr4-NHR-F	TN1950	5'- TATGGAGATGTCTCTGCTTTTTTTGCCGCCTCCTGTTCACC
clr4-NHR-R	TN1951	5'- GGTGAACAGGAGGCGGCAAAAAAAGCAGAGACATCTCCATAG
clr4-2	HM802	5'- GTCAGTGCCTCGTTCTC
mlo3-1	TN1747	5'- TCTGTTGCACTGAATCGTGC
mlo3-5	TN1751	5'- TCATCCAAACAAGCCGTGCC
mlo3-KA-R	TN1914	5'- CGTGTAGTCGCCCGCGCGGAAGATTTGGCGCCATTCTTGC
mlo3-KA-F	TN1913	5'- GCCAAATCTTCCGCGCGGGCGACTACACGCCGCCGTAGAAC
mlo3-KR-R	TN1925	5'- CGTGTAGTCCTCCGCCTGGAAGATTTGGCGCCATTC
mlo3-KR-F	TN1924	5'- GCCAAATCTTCCAGGCGGAGGACTACACGCCG
mlo3-4	TN1750	5'- TAACAGTAGCCGAAGCTACC
dh-1	TN370	5'- TGTCTCCATGTTGTTCGG
dh-2	TN371	5'- ACGCCCATTCATCAAGC
otr3-2	TN1704	5'- CGACAACAAAGCGACAATAGCAGTC
imr3-XhoI-R	TN2193	5'- TTAACAGGTCTCGAGGCCCAATGG
adl1-F	TN2233	5'- GTCTAGAATATGCCTCCCAAAAAGCG
adl1-R	TN2234	5'- TTTACGGTTCTGGGCCCCATTACCG
act1-F	TN2207	5'- GTACATTGCACCACTTCCGC
act1-R	TN2208	5'- AATAGGGACACGCGAGTTGC

Supplementary Table 3. The sequence of the primers used in real time PCR.

primer	stock #	target site	sequences
RT-dg102-F	HM980	dg	5'- TTGCACTCGGTTTCAGCTAT
RT-dg102-R	HM981		5'- TGCTCTGACTTGGCTTGTCT
dh-F	TN1943	dh	5'- CAACAGTATGGGTATAGAAAGAAGAC
dh-R	TN1944		5'- TGCATGCAAGAAACTCCATAACTT
imr3-out-F1	TN2179	imr3	5'- TGTCCAATTCTAACCACTCTATTACGA
imr3-out-R1	TN2180		5'- CATCATCAGCAACTGTCATTCTCA
spbc713.06_F	TN33	*adl1*	5'- AAATATGGCGATCCAGGAGATG
spbc713.06_R	TN34		5'- GCTTAACGTGCGCACAGACA
act1(ORF)-F	HM3273	*act1*	5'- AGCGTGGTTATACTTTCTCTACT
act1(ORF)-R	HM3274		5'- GGAGGAAGATTGAGCAGCAG

Supplementary Methods

Western blotting

Yeast extracts were prepared in the presence of trichloroacetic acid as described previously[2], separated by 10% SDS-PAGE (37.5:1), and transferred onto polyvinylidene difluoride membranes (PerkinElmer Life Sciences, NEF1002001PK). To detect Flag-tagged proteins, anti-FLAG M2 antibodies (Sigma-Aldrich, F1804) (1:2000) and peroxidase AffiniPure HRP-conjugated goat anti-mouse IgG (Jackson ImmunoResearch Laboratories, 115-035-146) (1:10000) were used as the primary and secondary antibodies, respectively. The blots were developed with SuperSignal West Femto substrate (ThermoScientific, 34095), and exposed using ImageQuant LAS500 (GE Healthcare). The blots were re-hybridized using anti-Mcm6 antibodies[3] (2:1000) and peroxidase AffiniPure HRP-conjugated goat anti-rabbit IgG (Jackson ImmunoResearch Laboratories, 111-035-003) (1:10000) as the primary and secondary antibodies, respectively.

Chromosome loss assay

Yeast cells were incubated for 6–8 days on EMM plates, and 10 mL of EMM liquid medium was inoculated with a single colony from EMM plates. After 2 day incubation, cells were plated onto YNB+LUA and 5FOA+LA media. At 8 days after plating, the number of total colonies and that of Ura^{-} colonies were counted on YNB+LUA and 5FOA+LA plates, respectively. Ura^{-} colonies formed on 5FOA+LA plates were incubated on EMM+UA and EMM+LU plates to inspect Leu$^{+/-}$ and Ade$^{+/-}$, respectively. The number of Leu^{-} Ura^{-} Ade^{-} cells indicative of chromosome loss was obtained by subtracting the numbers of Leu^{+} Ura^{-} Ade^{+}, Leu^{+} Ura^{-} Ade^{-}, and Leu^{-} Ura^{-} Ade^{+} cells from that of Ura^{-} cells. Cells were grown at 30 °C.

Supplementary References

1. Sievers, F. et al. Fast, scalable generation of high-quality protein multiple sequence alignments using Clustal Omega. *Mol Syst Biol* **7**, 539 (2011).

2. Onaka, A.T. et al. Rad51 and Rad54 promote noncrossover recombination between centromere repeats on the same chromatid to prevent isochromosome formation. *Nucleic Acids Res* **44**, 10744-10757 (2016).

3. Ogawa, Y., Takahashi, T. & Masukata, H. Association of fission yeast Orp1 and Mcm6 proteins with chromosomal replication origins. *Mol Cell Biol* **19**, 7228-7236 (1999).
